# Chemogenomics identifies acetyl-coenzyme A synthetase as a target for malaria treatment and prevention

**DOI:** 10.1016/j.chembiol.2021.07.010

**Published:** 2022-02-17

**Authors:** Robert L. Summers, Charisse Flerida A. Pasaje, Joao P. Pisco, Josefine Striepen, Madeline R. Luth, Krittikorn Kumpornsin, Emma F. Carpenter, Justin T. Munro, De Lin, Andrew Plater, Avinash S. Punekar, Andrew M. Shepherd, Sharon M. Shepherd, Manu Vanaerschot, James M. Murithi, Kelly Rubiano, Aslı Akidil, Sabine Ottilie, Nimisha Mittal, A. Hazel Dilmore, Madalyn Won, Rebecca E.K. Mandt, Kerry McGowen, Edward Owen, Chris Walpole, Manuel Llinás, Marcus C.S. Lee, Elizabeth A. Winzeler, David A. Fidock, Ian H. Gilbert, Dyann F. Wirth, Jacquin C. Niles, Beatriz Baragaña, Amanda K. Lukens

**Affiliations:** 1 Department of Immunology & Infectious Diseases, Harvard T.H. Chan School of Public Health, Boston, MA 02115, USA; 2 Research School of Biology, Australian National University, Canberra, ACT 2601, Australia; 3 Department of Biological Engineering, Massachusetts Institute of Technology, Cambridge, MA 02139, USA; 4 Wellcome Centre for Anti-Infectives Research, Drug Discovery Unit, Division of Biological Chemistry and Drug Discovery, University of Dundee, Dundee DD1 5EH, UK; 5 Department of Microbiology & Immunology, Columbia University Irving Medical Center, New York, NY 10032, USA; 6 Department of Pediatrics, University of California San Diego School of Medicine, La Jolla, CA 92093, USA; 7 Wellcome Sanger Institute, Hinxton CB10 1SA, UK; 8 Department of Chemistry, The Pennsylvania State University, University Park, PA 16802, USA; 9 Huck Center for Malaria Research, The Pennsylvania State University, University Park, PA 16802, USA; 10 Structural Genomics Consortium, Research Institute of the McGill University Health Centre, Montreal, QC H4A 3J1, Canada; 11 Department of Biochemistry and Molecular Biology, The Pennsylvania State University, University Park, PA 16802, USA; 12 Division of Infectious Diseases, Department of Medicine, Columbia University Irving Medical Center, New York, NY 10032, USA; 13 Infectious Disease and Microbiome Program, The Broad Institute, Cambridge, MA 02142, USA

**Keywords:** malaria, drug target identification, antimalarial, drug development, acetyl-CoA synthetase, *Plasmodium falciparum*, mechanism of action, histone acetylation

## Abstract

We identify the *Plasmodium falciparum* acetyl-coenzyme A synthetase (*Pf*AcAS) as a druggable target*,* using genetic and chemical validation. *In vitro* evolution of resistance with two antiplasmodial drug-like compounds (MMV019721 and MMV084978) selects for mutations in *Pf*AcAS. Metabolic profiling of compound-treated parasites reveals changes in acetyl-CoA levels for both compounds. Genome editing confirms that mutations in *Pf*AcAS are sufficient to confer resistance. Knockdown studies demonstrate that *Pf*AcAS is essential for asexual growth, and partial knockdown induces hypersensitivity to both compounds. *In vitro* biochemical assays using recombinantly expressed *Pf*AcAS validates that MMV019721 and MMV084978 directly inhibit the enzyme by preventing CoA and acetate binding, respectively. Immunolocalization studies reveal that *Pf*AcAS is primarily localized to the nucleus. Functional studies demonstrate inhibition of histone acetylation in compound-treated wild-type, but not in resistant parasites. Our findings identify and validate *Pf*AcAS as an essential, druggable target involved in the epigenetic regulation of gene expression.

## Highlights

• Mutations in *Pf*AcAS confer resistance to antiplasmodials MMV019721 and MMV084978
• MMV019721 and MMV084978 specifically inhibit *Pf*AcAS by competing with substrates
• cKD and IFA show *Pf*AcAS is an essential nuclear enzyme in blood-stage parasites
• *Pf*AcAS inhibitors deplete parasite acetyl-CoA and result in histone hypoacetylation

## Graphical abstract

**Figure:**
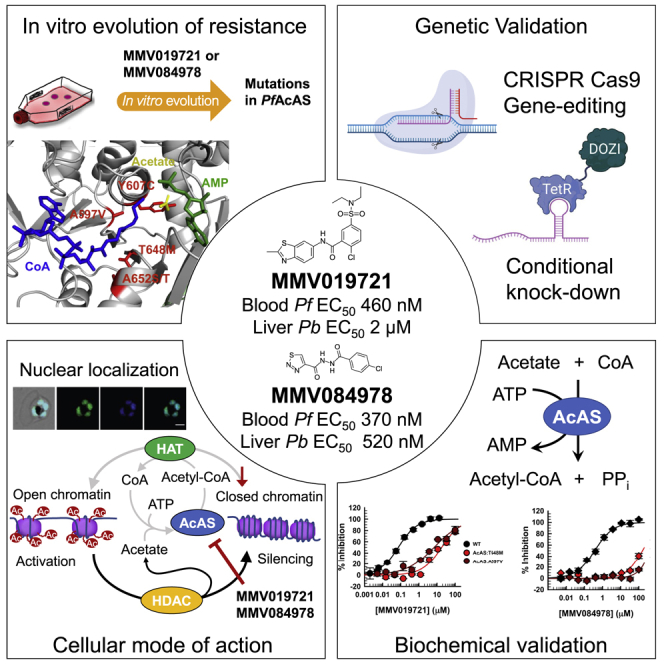

## Teaser

Using chemogenomics, Summers et al. identify PfAcAS as the target of two antimalarial compounds, MMV019721 and MMV084978. *Pf*AcAS is an essential, nuclear-localized enzyme responsible for acetyl-CoA biosynthesis in blood-stage parasites. Both compounds inhibit *Pf*AcAS competitively and act by depleting acetyl-CoA and disrupting histone acetylation in the parasite.

## Introduction

Despite significant gains in global malaria control efforts over the last decade, there are over 200 million new cases each year and nearly 500,000 deaths, primarily in young children in Africa (WHO, 2019). Resistance has emerged to most approved drugs and its spread may undermine the gains seen over the last decade. New drugs are urgently needed and a concerted effort by many in the field has resulted in the screening of over 5 million small molecules in phenotypic assays to assess parasite killing (Gamo et al., 2010; Guiguemde et al., 2010; Plouffe et al., 2008). These efforts have identified thousands of new chemical scaffolds. A major goal of new drug development for malaria is the discovery of compounds that kill parasites in multiple stages of the life cycle and, thus, could be used both in disease prevention and treatment. The Malaria Drug Accelerator (MalDA) consortium was convened to address the problem of target identification with an initial focus on chemogenomic approaches. We recently reported the screening of over 500,000 compounds for liver-stage activity and identification of hundreds of chemical scaffolds with liver-stage activity (Antonova-Koch et al., 2018). We subjected a subset of the available compounds to further analysis, including metabolomic profiling, additional phenotypic screening of blood-stage parasites, testing for cross-resistance, and *in vitro* evolution of drug resistance.

Many antimalarial molecules have been identified by whole-cell phenotypic screens, and most currently used antimalarial drugs were developed without precise knowledge of the drug target. However, many compounds with activity in phenotypic screens have subsequently been found to target one of a handful of common pathways in the parasite (e.g., hemoglobin degradation, mitochondrial function, and cellular homeostasis), that are susceptible to common resistance mechanisms (e.g., mutations in *Pf*CRT, *Pf*MDR1, *Pf*ATP4, or *Pf*DHODH) (Van Voorhis et al., 2016; Allman et al., 2016; Antonova-Koch et al., 2018; Creek et al., 2016). The development of drug candidates with novel modes of action requires the knowledge of the compound’s target. Furthermore, the identification and validation of new druggable targets enables modern therapeutic development strategies to be applied to malaria. Modern target-based drug discovery approaches can provide access to screening vast chemical diversity (for instance, via DNA-encoded libraries) to identify new chemical scaffolds, and to optimize potency and selectivity versus the human enzyme (via structure-guided drug design and biochemical assays of enzyme activity). Applying modern therapeutic development strategies to targets validated from phenotypic screens can complement earlier approaches and provides an accelerated path to malaria drug discovery.

MMV019721 and MMV084978 were chosen for further analysis because of promising characteristics: both compounds were active against multiple parasite life-cycle stages (liver and asexual blood stages) indicating the potential for both chemoprophylactic and curative therapeutic application, and neither compound was cross-resistant with previously identified targets, suggesting that they targeted a new pathway. Here, we present target identification and validation studies revealing the acetyl-coenzyme A synthetase (*Pf*AcAS; PF3D7_0627800) as the target of both MMV019721 and MMV084978. Acetyl-CoA synthetases catalyze the condensation of acetate and CoA into acetyl-CoA, which is a key cellular metabolite essential to cell survival. Here, we demonstrate that inhibition of *Pf*AcAS results in rapid cell death, disrupts intracellular acetyl-CoA levels, and provides strong evidence for the essentiality of this enzyme for parasite survival. *In vitro* biochemical validation studies confirm *Pf*AcAS as the target of MMV019721 and MMV084978. *Pf*AcAS is localized to the nucleus of the parasite, and mechanistic studies show that inhibition of *Pf*AcAS results in a reduction in histone acetylation. The work presented here identifying and validating *Pf*AcAS enables next-generation drug discovery for this essential enzyme.

## Results

### MMV019721 and MMV084978 are effective inhibitors of asexual- and liver-stage growth

**Figure 1:**
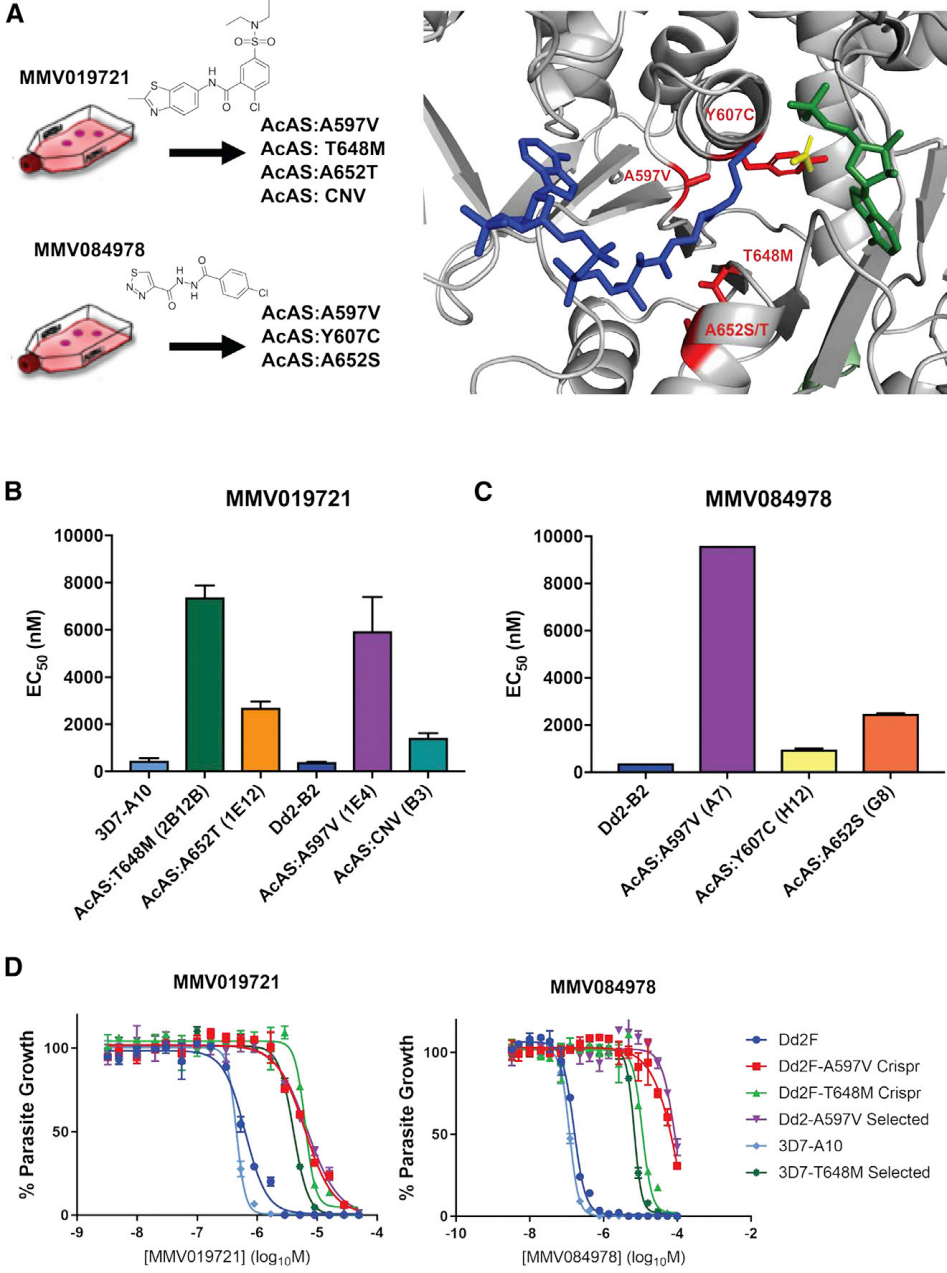
Mutations in the *P. falciparum* acetyl-CoA synthetase (*Pf*AcAS) confer resistance to MMV019721 and MMV084978 (A) Homology modeling of *Pf*AcAS reveal that mutations identified in parasites resistant to MMV019721 or MMV084978 line the predicted active site of the enzyme. (B and C) The *in vitro* susceptibility of representative drug-resistant cloned parasite lines identified as carrying mutations in *Pf*AcAS by WGS. Data represent the mean + standard deviation (SD) of four experiments conducted in triplicate for MMV019721, and the mean + SD of two experiments conducted in triplicate for MMV084978. (D) Representative dose-response assays for the 3D7 (light blue) and Dd2 (dark blue) parent lines, resistance-selected clones carrying A597V (purple) or T648M (light green), and CRISPR-Cas9 gene-edited parasites bearing A597V (red) or T648M (dark green). Shown is one representative biological replicate experiment run with technical triplicates. See also Figures S1 and S2, and Tables S1, S2, and S3.

Recent high-throughput whole-cell screens have identified a number of small molecules active against *Plasmodium* species (Antonova-Koch et al., 2018; Delves et al., 2018; Van Voorhis et al., 2016). Of particular interest are molecules that inhibit multiple life-cycle stages and can therefore function as both a curative therapeutic for blood-stage infection, but also act as prophylactic and/or transmission-blocking therapy. To identify molecules with prophylactic potential, recent screens have employed a *P. berghei* model system to screen for compounds with activity during liver-stage infection (Antonova-Koch et al., 2018). Two small molecules, MMV019721 and MMV084978, emerged from these screens and were selected for target identification efforts within the MalDA consortium (Figure 1A). MMV019721 has modest activity against blood-stage parasites *in vitro* (*P. falciparum* 3D7 half-maximal effective concentration [EC_50_] = 460 ± 100 nM) and against liver-stage *P. berghei* parasites (Pbluc EC_50_ = 2,100 ± 770 nM). MMV084978 demonstrated similar *in vitro* efficacy (*P. falciparum* Dd2 EC_50_ = 150 ± 36 nM) with increased liver-stage potency (Pbluc EC_50_ = 520 ± 210 nM). Neither molecule was active against HepG2 cells, indicating a promising lack of mammalian cell cytotoxicity (HepG2 EC_50_ > 50 μM for both compounds).

### MMV019721 and MMV084978 target acetyl-coA biosynthesis

A robust strategy for discovering the targets of cell-active compounds is *in vitro* evolution of resistance and whole-genome analysis, which has successfully identified the targets of many novel antimalarials discovered in phenotypic screens (Luth et al., 2018). To identify the target of MMV019721 and MMV084978, we conducted *in vitro* evolution experiments to select for resistant parasites by either a continuous or pulsed exposure to 3× or 4× EC_50_ concentrations of each compound (Table S1). Across 12 independent selections with MMV019721, bulk parasite populations with 3- to 18-fold resistance were obtained, and clones were isolated by limiting dilution. The resistant clones isolated upon exposure to MMV019721 showed an average 12-fold shift in the EC_50_ (range: 3.5- to 21-fold) (Figure 1B; Table S1). Whole-genome sequencing of 14 clones (1 or 2 each from 9 independent selections) revealed that all resistant lines carried single-nucleotide variants in a common gene, *PF3D7_0627800*, which codes for a putative acetyl-CoA-CoA synthetase (*Pf*AcAS) (Figure 1A; Tables S1 and S2). Five clones harbored a mutation resulting in an A597V amino acid change with an average 15-fold shift in EC_50_ over wild-type parasites (Dd2 EC_50_ = 400 ± 72 nM, *Pf*AcAS:A597V-resistant clone EC_50_ = 7,700 ± 570 to 11,000 ± 1,300 nM; Figure 1B; Table S1). Six clones carried a T648M mutation and a slightly higher resistance phenotype of 17- to 21-fold increase over wild-type (*Pf*AcAS:T648M EC_50_ = 6,800 ± 420 to 8,400 ± 1,400 nM; Figure 1B; Table S1). Two clones with low levels of resistance (∼3.5-fold over wild-type parasites) were found to possess amplification across a region of chromosome 6 containing *Pf*AcAS and three other genes: the ribonuclease P protein subunit p29 (PF3D7_0627900), 6-pyruvoyltetrahydropterin synthase (PF3D7_0628000), and the HECT-domain ubiquitin transferase (PF3D7_0628100) (Figure S1; Table S3).

Selections with MMV084978 yielded four clones ranging in resistance phenotype from 2.4- to 25-fold over wild-type parasites (Figure 1C; Table S1). Individual clones had one of three different mutations in the *Pf*AcAS gene, resulting in either an A597V, Y607C, or A652S amino acid change (Figure 1A; Table S2). Interestingly, the A597V mutation was identified in highly resistant lines in both compound selections, and similar mutations at position 652 (A652T or A652S in MMV019721 and MMV084978 selections, respectively) were seen in lines with intermediate levels of resistance to both compounds.

A further four clones with low-level resistance to MMV019721 (2- to 3-fold) lacked mutations in *Pf*AcAS but were found to possess mutations in acyl-CoA synthetase 11 (ACS11; T767I, S74L, D269G, and an indel L24-D30; Figure S2; Tables S1 and S2). None of the resistance-associated mutations in ACS11 occurred near the predicted active site of the protein (Figure S2A). Mutations in ACS11 have previously been associated with resistance to CoA-biosynthesis targeting antiplasmodial pantothenamides (Schalkwijk et al., 2019), and other structurally unrelated antiplasmodial compounds MMV019719 and MMV665924 (Cowell et al., 2018), suggesting that this locus may be a non-specific drug resistance mediator.

### Metabolomic profiling implicates PfAcAS inhibition in drug mode of action

**Figure 2:**
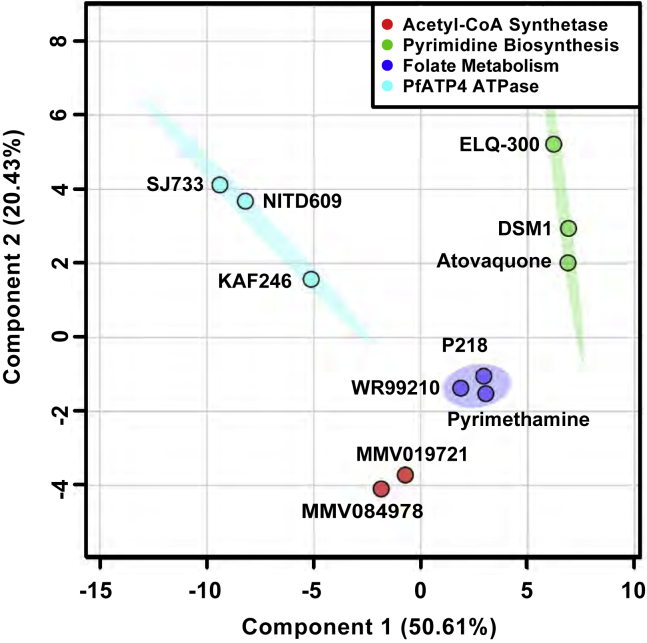
MMV019721 and MMV084978 induce unique cellular metabolomic profiles in *P. falciparum* parasites upon drug exposure Principal-component analysis (PCA) plot of the metabolic profiles of parasites treated with MMV019721 or MMV084978, in comparison to those of antiplasmodial compounds known to target mitochondrial function (atovaquone, ELQ-300, and DSM1, green), folate biosynthesis (pyrimethamine, P218, and WR99210, blue), or ion homeostasis (*Pf*ATP4-SJ733, NITD609, or KAF246, cyan) (Allman et al., 2016). PC1 represented 50.6% variance while PC2 represented 20.4% of the variance between all compounds. Principal components were calculated using the log2 fold-change in abundance of 98 soluble metabolites caused by test compounds relative to untreated parasite controls. PCA was conducted with MetaboAnalystR (Chong et al., 2019; Chong and Xia, 2018). See also Figure S3 and Table S4.

To confirm our *in vitro* evolution results indicating that these molecules both target *Pf*AcAS, we profiled the cellular metabolomic response to drug exposure for each compound and evaluated whether similar metabolic signatures were observed. Previous studies have identified distinct metabolomic profiles for antimalarials of known mechanism of action (Allman et al., 2016). Magnetically purified 3D7 parasites were incubated in the presence of each drug at 10-fold their reported EC_50_ value for 2.5 h (Allman et al., 2016). Principal-component analysis of metabolite profiles (Chong et al., 2019; Chong and Xia, 2018) indicates that the metabolic response to these compounds is distinct from known antimalarial drug responses, such as mitochondrial inhibitors (e.g., atovaquone, ELQ-300, and DSM1) or folate biosynthesis inhibitors (e.g., pyrimethamine, P218, and WR99210), and *Pf*ATP4 inhibitors (e.g., SJ733, NITD609, and KAF246) (Figures 2 and S3). Full analysis of the metabolic profile defined the major effects of MMV019721 and MMV084978 as a significant decrease in the levels of acetyl-CoA, N-carbamoyl-L-aspartate, dihydroorotate, and orotate (Figures S3C and S3D; Table S4). While not statistically significant, general nucleotide levels were increased in compound-treated samples (Figure S3D) and, when combined with the significant decreases in acetyl-CoA, N-carbamoyl-L-aspartate, dihydroorotate, and orotate, provided a unique metabolic profile (Figures 2 and S3) for both compounds.

### Allelic replacement studies demonstrate that PfAcAS mutations are sufficient for resistance to MMV019721 and MMV084978

To investigate the contributions of specific alleles, we introduced individual resistance mutations into an otherwise wild-type genetic background using CRISPR-mediated homology-driven repair with donor repair templates. The A597V and T648M *Pf*AcAS alleles were introduced into Dd2 parasites and their resistance phenotypes assessed. Both edited cell lines recapitulated the phenotype of the respective drug-selected clones (Figure 1D; Table 1), validating these mutations as sufficient to confer resistance to MMV019721 and MMV084978. Cross-resistance experiments further demonstrated that either A597V or T648M mutation was sufficient to confer cross-resistance to both compounds. This provides further evidence that *Pf*AcAS is the molecular target of these compounds.

**Table 1:** Dose-response phenotype of CRISPR-edited cell lines

Compound	3D7-A10 parent EC_50_ ± SD^a^ (nM)	Dd2-B2 parent EC_50_ ± SD^a^ (nM)	Dd2:A597V (CRISPR edited) EC_50_ ± SD^a^ (nM)	Dd2:T648M (CRISPR edited) EC_50_ ± SD^a^ (nM)
MMV019721	400 ± 58 (n = 3)	400 ± 72 (n = 4)	6,200 ± 94 (n = 4)	8,200 ± 1,700 (n = 3)
MMV084978	110 ± 28 (n = 3)	150 ± 36 (n = 4)	43,000 ± 6,800 (n = 3)	12,000 ± 1,200 (n = 4)
Mefloquine	18 ± 4.6 (n = 5)	15 ± 5.7 (n = 4)	31 ± 17 (n = 4)	20 ± 11 (n = 4)
Atovaquone	0.42 ± 0.05 (n = 3)	0.30 ± 0.13 (n = 4)	0.55 ± 0.35 (n = 4)	0.69 ± 0.38 (n = 4)
Dihydroartemisinin	2.6 ± 1.6 (n = 3)	4.6 ± 3.0 (n = 5)	5.6 ± 3.5 (n = 4)	3.7 ± 3.0 (n = 4)
Amodiaquine	7.4 ± 1.8 (n = 5)	19 ± 6.0 (n = 4)	21 ± 2.5 (n = 4)	20 ± 8.6 (n = 4)

Data presented as mean ± SD with n bioreplicate assays.

a SD, standard deviation.

### PfAcAS knockdown results in parasite growth arrest and demonstrates differential sensitivity to MMV019721 and MMV084978

**Figure 3:**
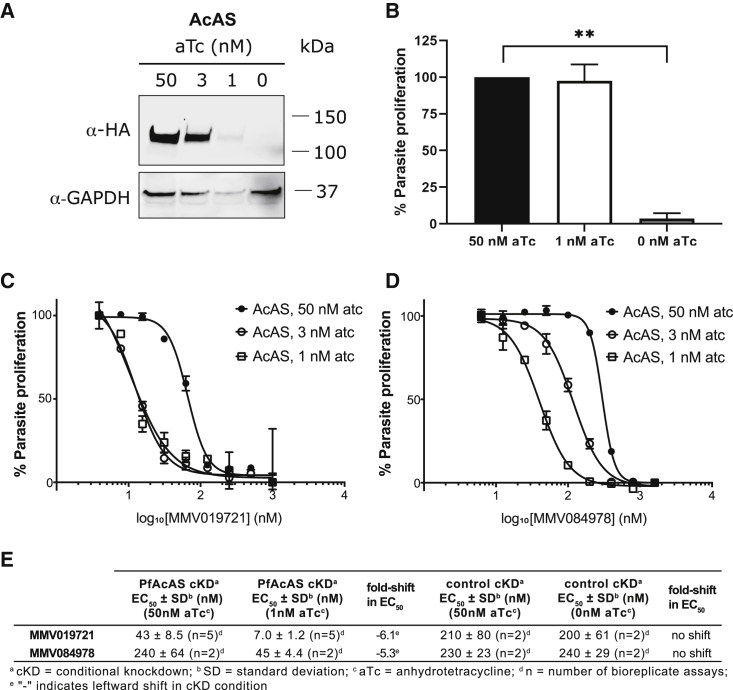
Conditional knockdown of *Pf*AcAS inhibits parasite growth and sensitizes parasites to MMV019721 and MMV084978 (A) Conditional knockdown (cKD) of *Pf*AcAS expression at reduced concentrations of aTc (3, 1, or 0 nM) over 72 h. (B) Parasite growth over 72 h is inhibited under cKD of *Pf*AcAS. Shown are the average results and SD of two independently repeated experiments with technical replicates. ∗∗p < 0.01, Student's t test compared with 50 nM aTc condition. (C and D) Susceptibility to MMV019721 and MMV084978 was increased under conditions of reduced *Pf*AcAS expression. Shown is one biological replicate run in triplicate. (E) Average EC_50_ ± SD of parasite susceptibility to MMV019721 and MMV084978 for *Pf*AcAS cKD and control YFP cKD lines under knockdown conditions.

It is possible that the mutations we discovered and genetically validated encode drug resistance genes rather than mutations that directly impede the target-inhibitor interaction. To assess whether the *Pf*AcAS enzyme is critical for parasite survival and whether it is a candidate for therapeutic inhibition, we utilized the anhydrotetracycline (aTc)-controlled TetR/DOZI–3′ UTR RNA aptamer system (Ganesan et al., 2016; Nasamu et al., 2020) to conditionally regulate the expression levels of *Pf*AcAS in the context of its native promoter. To assess the knockdown of the modified parasites, V5 and 2xHA epitope tags were fused directly downstream of the gene. Immunoblotting confirmed expression of an expected 117.9 kDa protein in the presence of aTc (Figure 3A). In contrast, aTc withdrawal substantially reduced *Pf*AcAS levels (Figure 3A), demonstrating that efficient knockdown had been achieved.

To determine if *Pf*AcAS is required for survival of intra-erythrocytic-stage parasites, cultures were maintained in the presence (50 and 3 nM) and absence of aTc. Analysis of growth over two replicative cycles revealed that, while parasites maintained in the presence of aTc were able to progress through the life cycle, aTc withdrawal resulted in substantial growth arrest (Figure 3B). Together, our data provide focused validation of the essentiality of *Pf*AcAS for parasite growth, and these are consistent with recent genome-wide piggyBAC insertion mutagenesis (Zhang et al., 2018) and knockout screens in *P. berghei* (Bushell et al., 2017) classifying AcAS as essential.

As a further validation of this target, we determined the sensitivity of the conditional knockdown parasites to MMV019721 and MMV084978. Reduction in *Pf*AcAS expression (low aTc) resulted in hypersensitivity to both MMV019721 and MMV084978, reflected by a leftward shift in dose-response curves compared with standard aTc conditions (Figures 3C and 3D). In contrast to the results observed with *Pf*AcAS, conditional knockdown of *Pf*ACS11 did not increase parasite sensitivity to either MMV019721 or MMV084978 (Figures S2C and S2D). These findings are consistent with MMV019721 and MMV084978 interacting directly with the *Pf*AcAS protein, while *Pf*ACS11 likely functions as a resistance mechanism for these compounds.

### Compounds directly and selectively inhibit PfAcAS activity *in vitro*

Acetyl-CoA synthetase enzymes catalyze the formation of acetyl-CoA from acetate and CoA in an ATP-dependent manner. Homology modeling of the *P. falciparum* protein using the crystal structure of acetyl-CoA synthetase from *Cryptococcus neoformans* H99 (PDB: 5U29) revealed that the resistance-conferring mutations cluster around the active site of the enzyme and, in particular, the predicted CoA binding site (Figure 1A). Mutations conferring the highest levels of resistance to both compounds, T648M and A597V, are predicted to occur close to the reactive center of the enzyme and face the CoA binding site from opposing surfaces of the protein. Together, these findings suggest that mutations in *Pf*AcAS may confer resistance to the compounds by preventing drug competition with substrate and alleviating inhibition of *Pf*AcAS function.

**Figure 4:**
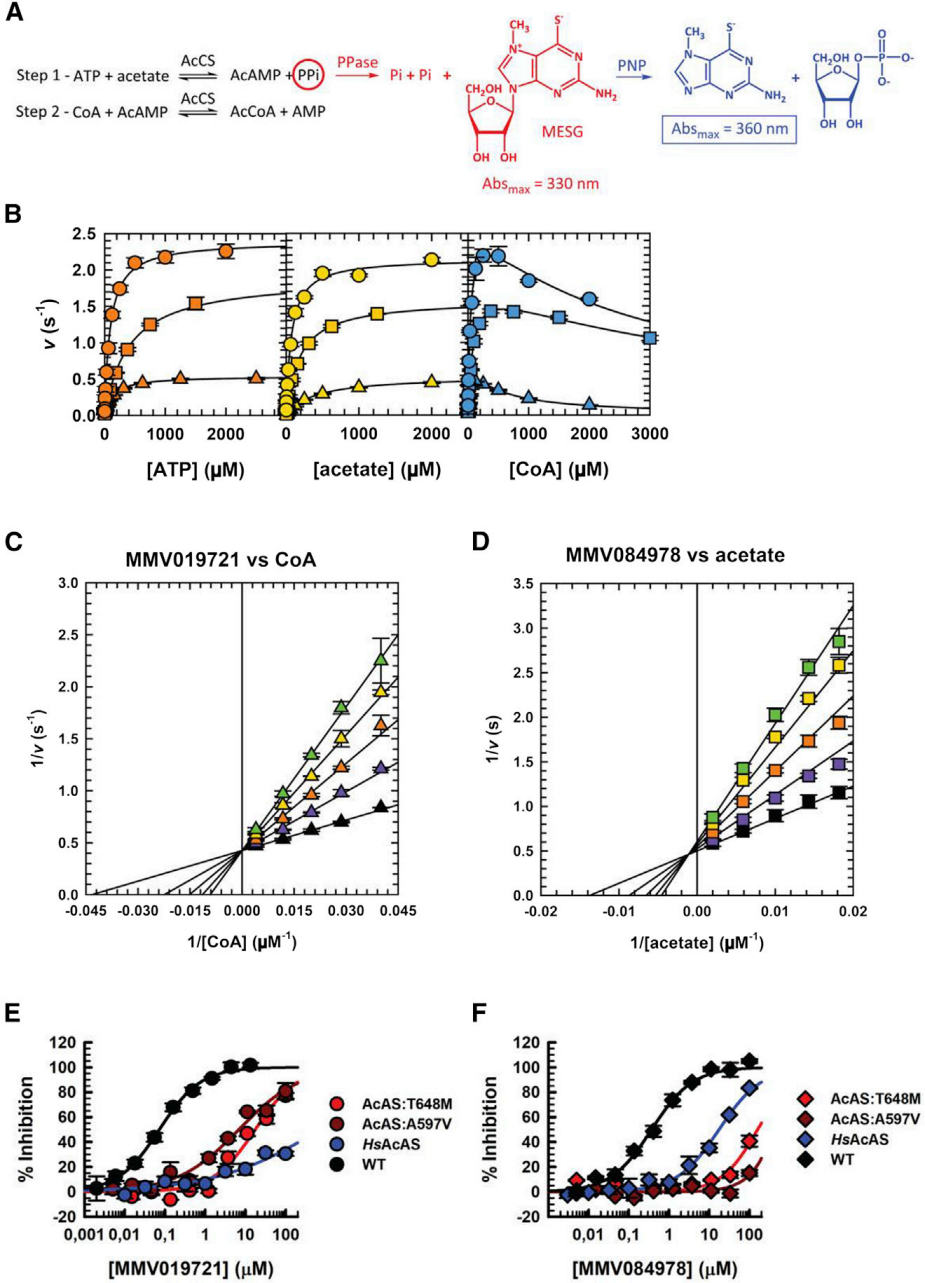
*Pf*AcAS steady-state kinetics and inhibition by MMV019721 and MMV084978 (A) *Pf*AcAS reaction mechanism and EnzChek assay readout. (B) Steady-state kinetics of *Pf*AcAS WT (circles), A597V (triangles), and T648M (squares). Saturation curves for ATP (orange), acetate (yellow), and CoA (blue). Error bars indicate the SD, n = 3. Lines are the best fit to Equations 1 (ATP and acetate) and 2 (CoA) in the STAR Methods. (C) Double-reciprocal plot illustrating the linear competitive inhibition pattern obtained when varying the concentration of MMV019721 at fixed variable concentrations of CoA and saturating concentrations of ATP and acetate. Points are data obtained with 0 (black), 20 (purple), 40 (orange), 60 (yellow), and 80 nM (green triangles) of MMV019721. The error bars indicate the SD, n = 3. Lines are the best fit of the entire dataset to Equation 4. (D) Double-reciprocal plot illustrating the linear mixed inhibition pattern obtained when varying the concentration of MMV084978 at fixed variable concentrations of acetate and saturating concentrations of ATP and CoA. Points are data obtained with 0 (black), 20 (purple), 40 (orange), 60 (yellow), and 80 nM (green squares) of MMV084978. The error bars indicate the SD, n = 3. Lines are the best fit of the entire dataset to Equation 5. Saturation curves for MMV019721 (E) and MMV084978 (F) against *Pf*AcAS WT (black symbols), A597V (dark red symbols), T648M (red symbols), and *Hs*AcAS (blue symbols). Error bars indicate the SD, n = 3. Lines are the best fit to Equation 3 and a linear fit for MMV084978 against *Pf*AcAS A597V (dark red line). See also Figures S4 and S5 and Tables S5 and S6.

To test this hypothesis, full-length recombinant wild-type *Pf*AcAS, T648M, and A597V proteins were expressed in Sf9 insect cells and purified (Figure S4A), and their activities studied using the EnzChek assay which measures inorganic phosphate originated by the breakdown of the pyrophosphate released in the first step of the *Pf*AcAS reaction (Webb, 1992) (Figures 4A and S5A). For the wild-type protein, *K*_m_ values of 93, 72, and 42 μM were obtained for ATP, acetate, and CoA, respectively (Figure 4B; Table S5). These values are in close relation to those observed for the *Salmonella enterica* (Reger et al., 2007), *Mycobacterium tuberculosis* (Noy et al., 2014), and *Homo sapiens* (Luong et al., 2000) AcAS orthologs. Inhibition of wild-type *Pf*AcAS by MMV019721 and MMV084978 was tested in the presence of saturating concentrations of all three substrates, ATP, acetate, and CoA, resulting in IC_50_ values of 73 ± 4 and 370 ± 36 nM, respectively (Figures 4B, 4E, and 4F; Table 2).

**Table 2:** Inhibition of PfAcCS WT, T648M and A597V, and HsAcAS by MMV019721 and MMV084978

Parameter	AcAS	MMV019721	MMV084978
IC_50_ (μM)	WT	0.073 ± 0.004	0.37 ± 0.04
	T648M	18 ± 3.2	>100
	A597V	6.9 ± 1.2	>100
	HsWT	>100	18 ± 1.9
Hill	WT	0.85 ± 0.04	0.85 ± 0.06
	T648M	0.81 ± 0.12	N/A^a^
	A597V	0.58 ± 0.05	N/A^a^
	HsWT	N/A^a^	0.85 ± 0.07

Data presented as mean ± SD.

a N/A, not applicable.

To study the mechanisms of inhibition by MMV019721 and MMV084978, single-inhibition measurements were performed at fixed saturating concentrations of two of the substrates, and variable concentrations of the third substrate. Under our experimental conditions, results for MMV019721 showed a linear uncompetitive inhibition versus ATP and acetate with *K*_i_ = 200 ± 8 and 150 ± 10 nM, respectively (Figures S5C and S5D; Table S6), and linear competitive inhibition against CoA with *K*_i_ = 22 ± 1 nM (Figures 4C and S5E; Table S6). These results suggest that MMV019721 inhibits *Pf*AcAS by preventing CoA from binding to the active site. Results for MMV084978 showed linear uncompetitive inhibition versus ATP and CoA, with *K*_i_ = 680 ± 26 and 690 ± 24 nM, respectively (Figures S5F–S5H; Table S6), and linear mixed inhibition against acetate with *K*_is_ = 30 ± 3 nM and *K*_ii_ = 390 ± 110 nM (Figures 4D and S5G; Table S6). These results suggest that MMV084978 inhibits *Pf*AcAS mostly by preventing acetate from binding to the active site; however, at higher concentrations, it can also inhibit the enzyme in the presence of acetate, suggesting that MMV084978 is most likely not occupying the acetate binding site.

Introduction of the A597V mutation increased the *K*_*m*_ of the protein for acetate by 5.5-fold (to 400 ± 12 μM), but had little or no effect on other substrates, while the T648M mutation increased the *K*_*m*_ for ATP by 4.2-fold to 400 ± 17 μM (Figure 4B; Table S5). The potency of both inhibitors was reduced against *Pf*AcAS A597V and T648M, with the IC_50_ value for MMV019721 increasing by 94-fold to 6.9 ± 1.2 μM, and 246-fold to 18 ± 3.2 μM for A597V and T648M, respectively (Figure 4E; Table 2). The IC_50_ value for MMV084978 increased to over 100 μM for both A597V and T648M (50% inhibition could not be obtained at 100 μM MMV084978, >271-fold change) (Figure 4F; Table 2). These results are consistent with parasite susceptibility observations in which the T648M mutation conferred higher levels of resistance to MMV019721, and A597V conferred higher level resistance to MMV084978 (Figure 1C). The effect of the A597V mutation on the acetate *K*_m_ value is also in line with the higher fold change observed in the inhibition of *Pf*AcAS A597V by MMV084978, which is mostly competitive against acetate.

To assess the selectivity of these two compounds for the *Plasmodium* enzyme, full-length recombinant wild-type *Hs*AcAS was expressed and purified (Figure S4B), and the activity was studied using a mass spectrometry detection assay (Rapid Fire), which measures the production of acetyl-CoA. Following the determination of the steady-state kinetic parameters (Figure S5I; Table S5), which were in close correlation to values in the literature (Luong et al., 2000), inhibition of wild-type *Hs*AcAS by MMV019721 and MMV084978 was tested in the presence of the approximate K_m_ for each substrate, resulting in an IC_50_ value over 100 μM for MMV019721 and 18 μM for MMV084978 (Figures 4E and 4F; Table 2).

These results further support *Pf*AcAS as the target of both molecules. Moreover, both these molecules possess drug-like properties and are amenable to medicinal chemistry optimization, adding confidence that this enzyme is a druggable target in *Plasmodia* and can be selectively inhibited.

### *Pf*AcAS localizes to the nucleus and plays a role in histone acetylation

**Figure 5:**
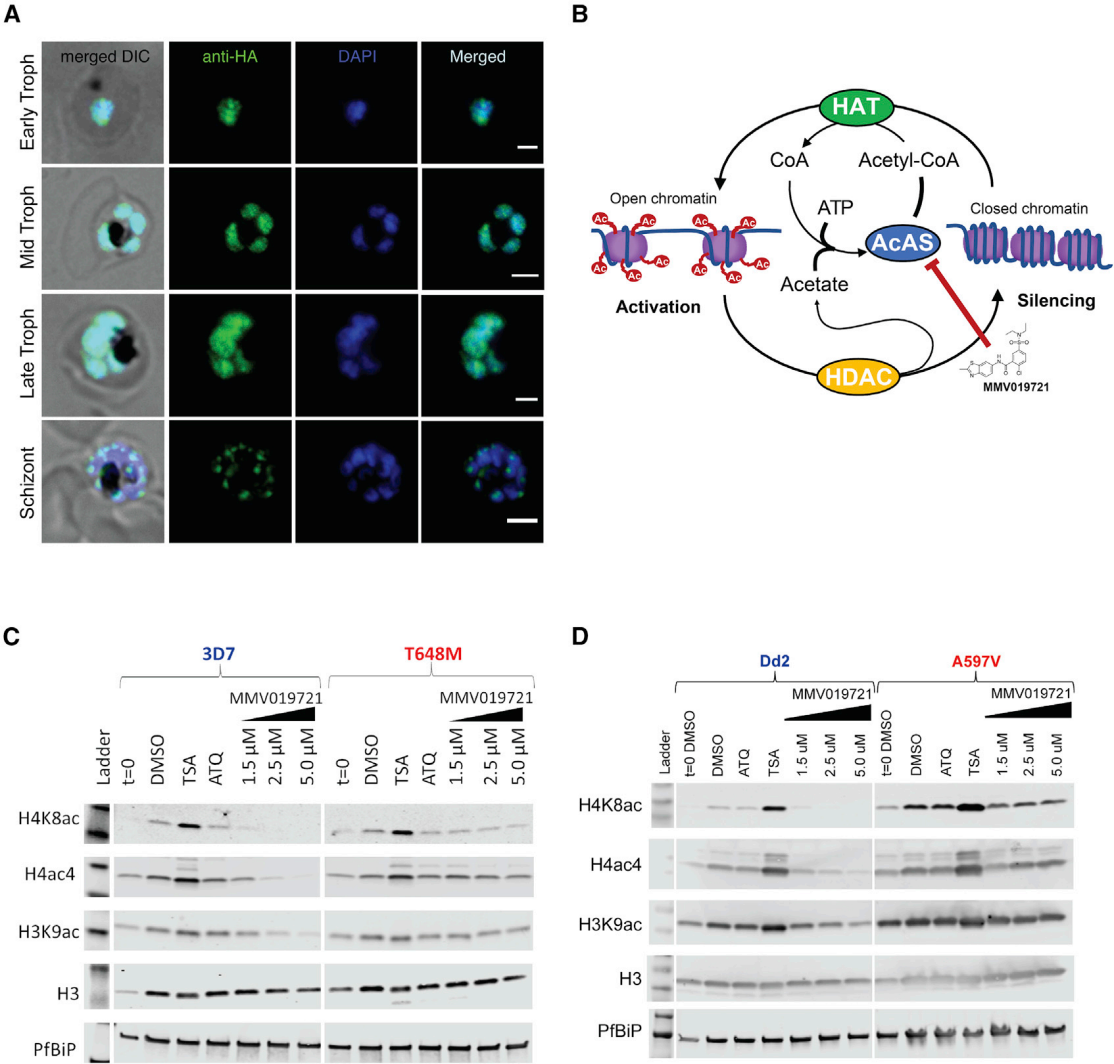
*Pf*AcAS localizes to the nucleus and its inhibition affects histone acetylation state (A and B) (A) Immunofluorescence localization of *Pf*AcAS using an HA-epitope-expressing construct throughout the life cycle of the parasite. Shown are representative images of early-, mid-, and late-stage trophozoite and schizont-stage parasites from a mixed-stage culture. *Pf*AcAS-HA co-localized with DAPI DNA stain throughout the majority of the parasite life cycle, consistent with nuclear localization of *Pf*AcAS. (B) Schematic representation of the proposed function of *Pf*AcAS in the maintenance of acetyl-CoA available for histone acetylation by histone acetyltransferases (HAT), from free acetate generated by the activity of histone deacetylases (HDAC). (C) Dose-dependent reduction of histone acetylation by MMV019721 in wild-type 3D7 parasites but not in *Pf*AcAS-T648M carrying drug-resistant parasites. Shown are representative western blots of a single experiment. (D) Dose-dependent reduction in histone acetylation by MMV019721 in Dd2 parent and not in A597V mutant parasites. Shown are representative western blots of a single experiment. See also Figures S6 and S7.

In other eukaryotes, subcellular localization of acetyl-CoA synthetase isoforms influences their role in maintaining acetyl-CoA pools for downstream cellular functions. To better understand the role of *Pf*AcAS, we determined the intracellular localization of the *Pf*AcAS protein by immunofluorescence assay (IFA) using an anti-HA antibody to probe for the epitope-tagged protein. Figure 5A shows overlap of the HA signal with the nuclear marker, DAPI, throughout the asexual developmental cycle, except in late-stage parasites where labeling was observed in punctate structures associated with the nucleus.

The localization of *Pf*AcAS to the nucleus suggested that *Pf*AcAS may play a role in the recycling of acetyl-CoA from CoA and acetate produced during the dynamic regulation of histone acetylation by histone acetyltransferases (HATs) and deacetylases (HDACs). Inhibition of this recycling function could deplete the nuclear store of acetyl-CoA, leading to hypoacetylation of histone proteins (Figure 5B). To test this, 24–32 h trophozoite-stage parasites were exposed to 1.5, 2.5, or 5 μM MMV019721 for a period of 3 h before isolation and analysis of parasite lysates by semiquantitative immunoblot. In wild-type 3D7-A10 parasites, increasing concentrations of MMV019721 caused a dose-dependent reduction in acetylation of the H4K8ac, H3K9ac, and tetra-acetylated H4 (H4Ac4) histone markers relative to DMSO-treated controls (Figures 5C and S6). The effect was most pronounced for H4K8ac, which is the primary component of H4 acetylation and is a highly dynamic marker during the blood-stage of *P. falciparum* (Gupta et al., 2017). There was no effect on histone acetylation by atovaquone, an antimalarial compound with a distinct mode of action, while the HDAC inhibitor trichostatin A (TSA), caused a significant increase in histone acetylation across all acetyl-histone markers, consistent with previous studies (Andrews et al., 2012; Gupta et al., 2017) (Figures 5C, 5D, and S6). There was no measurable effect of these treatments on total H3 abundance.

To determine whether *Pf*AcAS mutations, which confer resistance to MMV019721, also protect parasites from the effect of *Pf*AcAS inhibitors on histone acetylation, we conducted experiments in parallel with the 3D7-T648M parasite line. At concentrations resulting in significant histone hypoacetylation in wild-type parasites, there was little to no measurable effect on histone acetylation in the drug-resistant parasite line at all but the highest concentration of MMV019721 (Figures 5C and S6A). A similar dose-dependent reduction in histone acetylation was observed for MMV019721 in the Dd2 parent, and protection in the Dd2-A597V-resistant line (Figures 5D and S6B), and for MMV084978 in 3D7 and the 3D7-T648M lines (Figures S6C and S6D). Together, these results indicate that the effects of MMV019721 on histone acetylation were directly associated with *Pf*AcAS protein inhibition, and not with off-target effects.

In isobologram analyses, MMV019721 was strongly antagonistic with the HDAC inhibitor TSA, and to a lesser extent with the HAT inhibitor garcinol (Figure S7), suggesting that the function of HDAC and HAT enzymes is dependent on *Pf*AcAS function. Together, these findings are consistent with *Pf*AcAS playing an essential role in the maintenance of acetyl-CoA levels in the nucleus. Inhibition of *Pf*AcAS would prevent recycling of acetyl-CoA available for histone acetylation, thereby disrupting the epigenetic regulation of gene expression, and preventing growth in the blood stage of the parasite.

## Discussion

In this study, we have identified AcAS as a druggable target in *P. falciparum*. *In vitro* evolution of resistance experiments identified mutations in the *Pf*AcAS gene and allelic replacement of resistance mutations into an otherwise wild-type parasite background provided genetic validation and confirmed that each mutation in *Pf*AcAS was sufficient to confer resistance. Experiments using transgenic inducible knockdown parasites demonstrated that *Pf*AcAS is essential for asexual growth and the likely target of MMV019721 and MMV084978. Metabolic profiling identified changes in acetyl-CoA levels as a result of treatment and biochemical validation demonstrated that MMV019721 and MMV084978 directly inhibit *Pf*AcAS by preventing CoA and acetate binding, respectively. We also show that inhibition by MMV019721 and MMV084978 is highly selective for the *Plasmodium* enzyme compared with the human enzyme. Taken together, these data give excellent genetic and chemical validation of *Pf*AcAS. MMV019721 and MMV084978 are small molecules with drug-like properties, suggesting that it should be possible to develop small-molecule inhibitors of this enzyme as potential therapeutic agents. Immunolocalization studies indicate that *Pf*AcAS is primarily localized to the nucleus, while functional studies in whole cells revealed inhibition of histone acetylation in drug-treated wild-type cells with no or limited inhibition in parasites carrying either of the two major resistance mutations.

Acetyl-CoA is central to several cellular pathways, including the TCA cycle, and lipid and phospholipid synthesis, and it is important to consider the impact on these pathways (Pietrocola et al., 2015). Experimental work in other eukaryotes has documented disruption of each of these processes (Bulusu et al., 2017; Mashimo et al., 2014; Starai and Escalante-Semerena, 2004). In those systems, there are multiple AcAS genes encoding proteins with specific subcellular localizations associated with their primary functional role (Starai and Escalante-Semerena, 2004). In yeast, neither of the two AcAS genes is essential, but deletion of both is lethal (Takahashi et al., 2006). In *P. falciparum* there is a single, essential AcAS gene. Recent work described a series of panthothenamide antimetabolites that interfere with acetyl-CoA metabolism in *P. falciparum*, and cause depletion of the cellular acetyl-CoA pool (Schalkwijk et al., 2019). Metabolomic profiling of infected red cells treated with MMV019721 or MMV084978 similarly demonstrated a depletion of acetyl-CoA. Interestingly, in *P. falciparum,* previous metabolomics work has demonstrated that acetyl-CoA has two biosynthetic paths via a mitochondrial branched-chain keto acid dehydrogenase (BCKDH) complex or via acetyl-CoA synthetase (Cobbold et al., 2013). Our study and the work of Schalkwijk et al. (2019) demonstrate that *Pf*AcAS activity is essential, implying that acetyl-CoA derived from the BCKDH pathway cannot compensate for loss of *Pf*AcAS activity.

Mammalian orthologs of *Pf*AcAS have been shown to play a critical role in the regulation of epigenetic function, including in metabolic switching of cancer cells in response to anoxic stress, and in hippocampal cells associated with memory retention (Bulusu et al., 2017; Comerford et al., 2014; Li et al., 2017a, 2017b; Mews et al., 2017). Elegant work in yeast demonstrates the critical role for nuclear-localized acetyl-CoA synthetase in histone acetylation and the correlation with cell viability (Takahashi et al., 2006). There are relatively few transcription factors in the *P. falciparum* genome, and it has been suggested that epigenetic regulation may play an integral role in cell-cycle regulation in *Plasmodium* (Templeton et al., 2004; Balaji et al., 2005; Hollin et al., 2020). Recently, *Pf*AcAS has been identified as a putative member of a chromatin remodeling complex that is associated with the promoter region of highly transcribed genes, which also coincides with enrichment of H3K9 acetylation (Bryant et al., 2020). The involvement of *Pf*AcAS in this critical function could suggest a unique role in linking the metabolic state of the cell to epigenetic regulation in the parasite, and highlights enzymes involved in epigenetic regulation as an attractive target for antimalarial drug discovery.

It is interesting to note that, in a small subset of clones, mutations in the acyl-CoA synthetase *Pf*ACS11 were associated with low-level resistance to MMV019721. Mutations in this gene have previously been found in association with *Pf*AcAS mutations in parasites selected for resistance to the coenzyme A anti-metabolite-forming pantothenamides (Schalkwijk et al., 2019). Another study has also identified ACS11 as a potential resistance mediator to unrelated antiplasmodial compounds (MMV019719 and MMV665924) (Cowell et al., 2018). Conditional knockdown of ACS11 had no effect on parasite susceptibility to MMV019721 or MMV084978, and mutations in ACS11 did not localize to any clear functional domains within the protein, suggesting that ACS11 is not a primary target of these compounds. Together, the results of these studies suggest that ACS11 may be a mediator of resistance, but further experimental work will be needed to define the ACS11 function.

The work presented here clearly identifies *Pf*AcAS as the target of the small-molecule inhibitors MMV019721 and MMV084978. Single mutations in the gene are sufficient to confer resistance both in cell culture and when purified enzymes are profiled *in vitro*. Together, these observations provide good support for inhibitor potency having a direct effect on drug susceptibility in the parasite, and bodes well for medicinal chemistry efforts. Cytotoxicity studies in HepG2 cells and *in vitro* enzymatic assays testing the human ortholog *Hs*ACSS2 showed the potential for high selectivity between the parasite and host enzymes, increasing the promise of this target for antimalarial drug development. A working model for the mechanism of killing is that inhibition of *Pf*AcAS results in disruption of acetyl-CoA recycling in the nucleus leading to a reduction in histone acetylation. This, in turn, dysregulates the epigenetic program of the tightly controlled gene expression in *P. falciparum* and causing cell death.

## Significance

**Malaria is a global disease, which results in more than 200 million infections each year, and kills in excess of 300,000 people every year, predominantly children and pregnant women. The emergence and spread of drug resistance places many of the frontline antimalarials currently in use at risk, and there is an urgent need to refresh the antimalarial arsenal. Many of the antimalarial candidates currently under development act against a relatively narrow range of parasite targets, are susceptible to drug resistance via common pathways, or are only active against blood stages of the parasite. To** **support** **the goal of malaria control and eradication, it is crucial to identify new drug targets that have activity against multiple stages of the parasite, and thereby provide chemoprotective or transmission-blocking effects in addition to therapeutic activity. For these reasons, the identification and validation of novel druggable targets, particularly those important at multiple stages of parasite development, is a key priority for the field. Here, we applied an array of chemogenomic, metabolomic, genetic, and biochemical approaches to identify and validate the parasite’s acetyl-CoA synthetase (*Pf*AcAS) as the target of two compounds (MMV019721 and MMV084978) active against both liver and blood stages of the parasite. We also present evidence that PfAcAS inhibitors disrupt the regulation of histone acetylation, suggesting that *Pf*AcAS may represent an Achilles heel in the epigenetic regulation of parasite gene expression. Together, these studies validate *Pf*AcAS as a drug target suitable for malaria chemoprevention and treatment and provide a set of tools to** **support** **the target-based identification of novel and selective PfAcAS inhibitors.**

## STAR★Methods

### Key resources table

**Table:** 

REAGENT or RESOURCE	SOURCE	IDENTIFIER
**Antibodies**		

mouse anti-HA	Sigma	Cat#H3663; RRID: AB_262051
rabbit anti-GAPDH	Abcam	Cat#AB9485; RRID: AB_307275
anti-mouse horseradish peroxidase (HRP)-conjugated	Thermo Fisher Scientific	Cat#62-6520; RRID: AB_2533947
anti-rabbit horseradish peroxidase (HRP)-conjugated	Cell Signaling Technology	Cat#7074S; RRID: AB_2099233
Rabbit anti tetra-acetylated H4	Sigma-Aldrich	Cat#06-866; RRID: AB_310270
Rabbit anti-H4K8ac	Cell Signaling Technology	Cat#2594; RRID: AB_2248400
Rabbit anti-H3K9ac	Cell Signaling Technology	Cat#9649; RRID: AB_2248400
Rabbit anti- H3	Cell Signaling Technology	Cat#4499; RRID: AB_10544537
Rabbit anti-*Pf*BiP	(Shonhai et al., 2007)	N/A
800CW goat anti-rabbit IgG	Licor	Cat#926-32211; RRID: AB_621843
anti-mouse Alexa Fluor® 488-conjugated secondary antibody	Cell signaling Technology	Cat#4408S; RRID: AB_10694704
Mouse anti-His antibody	Pierce	Cat#MA1-21315; RRID: AB_557403

**Bacterial strains**		

*E. coli* DH10Bac	Invitrogen	Cat#10361012
*E. coli* XL1 cells	Agilent Technologies	Cat#50125058

**Chemicals, peptides, and recombinant proteins**		

SYBR Green I fluorescent dye	Invitrogen	Cat#S7563
MitoTracker Deep Red FM	Life Technologies	Cat#M22426
MMV084978	Medicines for Malaria Venture (Antonova-Koch et al., 2018)	N/A
MMV019721	Medicines for Malaria Venture (Duffy et al., 2017)	N/A
WR99210	Jacobus Pharmaceuticals	CAS 47326-86-3
Anhydrotetracycline hydrochloride	Sigma-Aldrich	Cat#37919; CAS 13803-65-1
Blasticidin S hydrochloride	RPI Corp	Cat#B12150-0.1; CAS 3513-03-9
Trichostatin A	Sigma-Aldrich	Cat#T8552; CAS 58880-19-6
Atovaquone	AK scientific	Cat#G211; CAS 95233-18-4
Chloroquine diphosphate salt	Sigma-Aldrich	Cat# C6628; CAS 50-63-5

**Critical commercial assays**		

Nextera XT kit	Illumina	Cat# FC-131-1024
DNeasy Blood and Tissue Kit	Quiagen	Cat#69506
SuperSignal® West Pico Chemiluminescent substrate	Thermo Fisher Scientific	Cat#PI34080
Expi293™ Expression System Kit	Thermo Fisher Scientific	Cat#A14635
Renilla-Glo(R) Luciferase Assay System	Promega	Cat#E2750
Maxiprep system	Qiagen	Cat#12163
EnzChek™ Phosphate Assay Kit	Thermo Fisher Scientific	Cat#E6646

**Deposited data**		

Whole Genome Sequencing data	NCBI Sequence Read Archive (SRA)	SRA BioProject Accession number: PRJNA684602
Metabolomics Data	National Metabolomics Data Repository (NMDR)	NMDR Project ID: PR001065
Crystal structure of acetyl-CoA synthetase from *C. neoformans* H99 used for PfAcAS modelling	RCSB Protein Data Bank	PDBID: 5U29
Crystal structure of the adenylation domain of carboxylic acid reductase of *Nocardia iowensis* in the adenylation conformation used for *Pf*ACS11 modelling	(Gahloth et al., 2017)	PDBID: 5MSD

**Experimental models: cell lines**		

*H. sapiens* Expi293 mammalian cells	Thermofisher	Cat# A14527
*S. frugiperda* Sf9 insect cells	Novagen	Cat#71104-M
NF54-PfACS11-cKD	This paper	N/A
Dd2-B2	(Cowell et al., 2018)	N/A
NF54-expressing Cas9- and T7 RNA polymerase	(Nasamu et al., 2020)	N/A
NF54-PfAcAS-cKD	This paper	N/A
Dd2-B2-A597V_crispr	This paper	N/A
Dd2-B2-T648M_crispr	This paper	N/A
See Table S2 for additional selected cell lines		

**Oligonucleotides**		

Oligos for sequencing of PfAcAS (See Table S7)	Integrated DNA Technologies	N/A
Oligos for CRISPR gene editing and plasmid construction (See Table S7)	ThermoFisher	N/A

**Recombinant DNA**		

pDC2-coCas9-U6.2-hDHFR plasmid	(Lim et al., 2016)	N/A
pDC2-coCas9-gRNA-hDHFR plasmid	(Lim et al., 2016)	N/A
pDC2-coCas9-AcAS-A597V-gRNA1-hDHFR	This paper	N/A
pDC2-coCas9-AcAS-T648M-gRNA1-hDHFR	This paper	N/A
pDC2-coCas9-gRNA2-hDHFR	This paper	N/A
pSN054	(Nasamu et al., 2020)	N/A
AcAS_pSN054	This paper	N/A
pET15b	Novagen	Cat#118754
pET15b-PPT343	This paper	N/A
pFastBac™-HT	Invitrogen	Cat#10584027
pFastBac-PPT358	This paper	N/A
pFastBac-PPT409	This paper	N/A
pFastBac-PPT410	This paper	N/A

**Software and algorithms**		

GATK HaplotypeCaller	(McKenna et al., 2010),	https://gatk.broadinstitute.org/hc/en-us
El-Maven LC/MS data processing software	El-Maven	https://resources.elucidata.io/elmaven
MetaboAnalystR 2.0	(Chong and Xia, 2018; Chong et al., 2019)	https://www.rdocumentation.org/packages/MetaboAnalystR/versions/2.0.0
I-TASSER pipeline	(Yang and Zhang, 2015; Zhang et al., 2017)	https://zhanglab.ccmb.med.umich.edu/I-TASSER/
PyMOL 2.2.3	Schrödinger, Inc.	https://pymol.org/2/
Benchling	Benchling	https://benchling.com/
Image Lab 5.2.0	Bio-Rad	https://www.bio-rad.com/en-us/product/image-lab-software?ID=KRE6P5E8Z
Image Studio ver 5.2	Li-Cor	https://www.licor.com/bio/image-studio/
ImageJ	NIH	https://imagej.nih.gov/ij/
Graphpad Prism 8.0	Graphpad Software Inc.	https://www.graphpad.com/scientific-software/prism/

### Resource availability

#### Lead contact

Further information and requests for resources and reagents should be directed to and will be fulfilled by the lead contact, Amanda Lukens (alukens@broadinstitute.org).

#### Materials availability

Plasmids and cell lines generated in this study are available upon request. Depending on the reagent and institution of origin, an MTA might be required.

#### Data and code availability

The genome sequence data generated during this study are available at the NCBI Sequence Read Archive (SRA), BioProject Accession number: PRJNA684602. The metabolite data generated during this study are available at the NIH Common Fund's National Metabolomics Data Repository (NMDR) website, the Metabolomics Workbench, https://www.metabolomicsworkbench.org, where it has been assigned Project ID: PR001065. The data can be accessed directly via it's Project https://doi.org/10.21228/M81Q3K. This work is supported by NIH grant U2C-DK119886.. This paper does not report original code. Any additional information required to reanalyze the data reported in this paper is available from the lead contact upon request.

### Experimental model and subject details

#### Parasite cell lines and culture

The 3D7-A10 clone has been used in previous *in vitro* drug selection and sequencing efforts (Cowell et al., 2018). Dd2 parasites were obtained from T. Wellems (NIAID, NIH). Dd2-B2 is a genetically homogeneous line that was cloned from Dd2 by limiting dilution in the Fidock lab. Parasite strains were cultured in 3-5% human O+ hematocrit in RPMI 1640 (Life Technologies) supplemented with 28 mM NaHCO_3_, 25 mM HEPES, and hypoxanthine (50 mg/mL). Depending on the parasite line, the media either contained gentamycin (25 μg/mL), or no antibiotic and was additionally supplemented with 0.5% AlbuMAX II (Life Technologies) and/or O+ human serum (heat inactivated and pooled). Blood and serum products were obtained from Interstate Blood Bank. Cultures were maintained at 37°C in 1.1% O_2_, 4% CO_2_, and 95% N_2_. Parasite populations were synchronized by 5% sorbitol treatment (Lambros and Vanderberg, 1979).

#### Insect cell line and culture

Sf9 cells were propagated in Gibco Sf-900 II SFM media supplemented with 2 mM L-glutamine and 100 U/ml penicillin/streptomycin. Sf9 cells were routinely maintained as shake flask cultures grown in 250 mL Nalgene Single-Use PETG Erlenmeyer flasks (ThermoFisher Scientific) stirred at 135 rpm and 27°C to a cell density of 15–20 × 10^5^ cells ml^−1^ and ≥95% cell viability.

### Method details

#### Dose-response assay

For *in vitro*–selected lines, drug susceptibility was measured by SYBR Green I–based assay (Johnson et al., 2007; Smilkstein et al., 2004). Ring-stage parasites were cultured for 72 hr at 1% hematocrit and 1% starting parasitemia in 384-well black clear-bottom plates containing test compounds plated in triplicate in 12-point serial dilutions. Lysis buffer (0.16% w/v saponin, 1.6% Triton X-100, 5 mM EDTA, and 20 mM Tris-HCl, pH 7.4) with SYBR Green I fluorescent dye (Invitrogen) was added, and fluorescence readings were taken (excitation at 494 nm, emission at 530 nm). Alternatively, Dd2-B2 ring-stage cultures at 0.3% parasitemia and 1% hematocrit were exposed for 72 hr to a range of ten drug concentrations that were 2-fold serially diluted in duplicates along with drug-free controls. Parasite survival was assessed by flow cytometry on an Accuri C6 (BD Biosciences) using SYBR Green and MitoTracker Deep Red FM (Life Technologies) as nucleic acid stain and vital dyes respectively. EC_50_ values were calculated using a nonlinear regression curve fit in Prism Software version 8 (GraphPad).

#### Resistance selection

3D7-A10 or Dd2-B2 clones were expanded to ∼1.5x10^7^ parasites per flask (3x 25 mL at 5% hematocrit, 4% parasitemia) and exposed to 3xEC_50_ concentrations of MMV019721 until cultures were smear-negative (∼5–7 days). Cultures were then maintained in the absence of drug until parasites were detected by microscopy (7–14 days), at which point parasite-positive flasks were split into two cultures and exposed to higher concentrations of compound (6xEC_50_) until no impaired growth was apparent, or were maintained in the absence of selection compound. Bulk cultures were tested for reduced drug susceptibility and individual clones were isolated by limiting dilution. MMV019721 resistant bulk cultures and clones were genotyped by whole-genome sequencing and confirmed by sanger-sequencing of the *Pf*AcAS locus (amplified using SeqF1 and SeqR4 primers) and the following primer pairs: SeqF1 CAATGAATAATTTGAAGAGTTATGG, SeqR1: CTGGGAACAAATATTTAAATGG, SeqF2: GGATATGTGTGGAATGATACAAAC, Seq R2: CCAGCTGTTGTATGTGCAAC, Seq F3: CCTGAACCTGATAAAAATAGCACAAGC, Seq R3 GAAATGAAACAACAGCTGCTTCAGC, SeqF4 CAGCAGAAATTGAACATGCACTAGTTC, Seq R4 TAGCGTCTCGAGTTATTTCTTAATTTCAATATGCTTTAACTTTTTTTTACA.

#### Library preparation and whole genome sequencing

Infected RBCs were washed with 0.05% saponin and genomic DNA was isolated from the parasites using a DNeasy Blood and Tissue Kit (Qiagen) according to the standard protocols. Sequencing libraries were prepared with the Nextera XT kit (Cat. No FC-131-1024, Illumina) via the standard dual index protocol and sequenced on the Illumina HiSeq 2500 in RapidRun mode to generate paired-end reads 100bp in length. Sequence data is available under BioProject Accession number: PRJNA684602 in the NCBI Sequence Read Archive. Reads were aligned to the *P. falciparum* 3D7 reference genome (PlasmoDB v13.0) using the previously described pipeline (Cowell et al., 2018). A total of 18 clones were sequenced to an average whole genome coverage of 52x, with an average of 97% of reads mapping to the reference genome (Table S1). Following alignment, SNVs and INDELs were called using GATK HaplotypeCaller and filtered according to GATK's best practice recommendations (McKenna et al., 2010). Variants were annotated using a custom SnpEff database and further filtered by comparing those from resistant clones to the parent clone, such that only a mutation present in the resistant clone but not the sensitive parent clone would be retained. CNVs were identified by differential Log2 copy ratio as described in the GATK 4 workflow. Briefly, read counts were collected across genic intervals for each sample. Copy ratios were calculated after denoising read counts against a strain-matched Panel of Normals comprised of non-drug-selected Dd2 or 3D7 parasite samples.

#### Metabolite extraction

The metabolite extraction protocol was performed as described elsewhere (Allman et al., 2016). Briefly, 1 mL of trophozoites that were magnetically purified to a concentration of 1×10^8^ parasites/mL were placed into wells with 4 mL of RPMI 1640 media and allowed to recover for one hour. A drug corresponding to the appropriate condition was added to each well to a final concentration of 10-fold above the EC_50_ of that compound. The culture was incubated for another 2.5 hr. The culture was centrifuged and quenched with 90% methanol containing 0.25 μM ^13^C,^15^N-Labeled-Aspartate, vortexed, and centrifuged. The supernatant was transferred to a new tube and dried for storage. Prior to analysis, the samples were resuspended to a final concentration of 1×10^8^ parasites/mL in a solution of 3% methanol:water containing 1 μM chlorpropamide as an internal standard.

#### Metabolomics LC/MS analysis

The chromatographic conditions used in this experiment were performed with slight modifications from previous methods (Zhang et al., 2018). Briefly, a Waters XSelect HSS C18 column was used on a gradient reverse-phase chromatographic configuration with 97:3 water:methanol with 10 mM tributylamine and 15 mM acetic acid (solvent A) and methanol (solvent B). A Thermo-Scientific Exactive Plus Orbitrap mass spectrometer was operated in negative mode for the detection of the metabolites. Peak picking and integration were performed using El-Maven LC/MS data processing software. The peak data were then exported to Excel where metabolite areas were normalized to the internal standards, blank subtracted, and averaged by condition.

#### Homology modeling studies

Homology models of *Pf*AcAS and *Pf*ACS11 were prepared using the I-TASSER pipeline (Yang and Zhang, 2015; Zhang et al., 2017) based on the solved *Cryptococcus neoformans* H99 structure for *Pf*AcAS (PDB:5U29) and the solved structure of the adenylation domain of carboxylic acid reductase of *Nocardia iowensis* in the adenylation conformation for *Pf*ACS11 (PDB:5MSD) (Gahloth et al., 2017). Hydrogen atoms were added to each model and Gasteiger charges calculated using AutoDockTools 1.56. Images were rendered using PyMOL 2.2.3 (Schrödinger, Inc.).

#### CRISPR/Cas9 editing of PfAcAS

Mutant *Pf*AcAS-A597V and *Pf*AcAS-T648M parasites were generated using CRISPR/Cas9. The guide RNAs for *Pf*AcAS-A597V (5′-GTTGTGTAGCAGATATAGGT-3′; 5′-GCAGATATAGGTTGGGTTAC-3′) and for *Pf*AcAS-T648M (5′-ATTAATGCTCTTAAGGCTGT-3′) were designed using Benchling (https://benchling.com/) to targeting sites in *Pf*AcAS that were 6-14 bp upstream of the A597 codon and 6 bp downstream of the T648 codon respectively. Complementary oligos encoding the gRNA sequences (5′- tattGTTGTGTAGCAGATATAGGT-3’ / 5’-aaacACCTATATCTGCTACACAAC-3’; 5’-tattGCAGATATAGGTTGGGTTAC-3’ / 5’-aaacGTAACCCAACCTATATCTGC-3’ and 5’- tattgATTAATGCTCTTAAGGCTGT-3’ / 5’-aaacACAGCCTTAAGAGCATTAATc-3’ ) were phosphorylated using T4 polynucleotide kinase and annealed (95°C for 5 min, then step down of 5°C/min to 25°C) prior to ligation into the BbsI-digested pDC2-coCas9-U6.2-hDHFR plasmid (Lim et al., 2016). The donor template for *Pf*AcAS-A597V was synthesized (ThermoFisher) as a 700 bp Genestring fragment spanning position 1468 to 2168 of the *Pf*AcAS gene, and the donor for *Pf*AcAS-T648M synthesized as a 829 bp fragment spanning position 1468 to 2297 and inserted into the AatII/EcoRI sites of the pDC2-coCas9-gRNA-hDHFR plasmid by Gibson assembly. Ring-stage parasites (Dd2) at 5% parasitemia were transfected by electroporation (BioRad Gene Pulser II at 0.310 kV and 950 μF) with 50 μg of each plasmid (plasmid 1: pDC2-coCas9-AcAS-A597V-gRNA1-hDHFR, plasmid 2: pDC2-coCas9-gRNA2-hDHFR, or plasmid 3: pDC2-coCas9-AcAS-T648M-gRNA1-hDHFR), and parasites selected for 10 days with 2.5 nM WR99210 (Jacobus Pharmaceuticals), followed by removal of drug pressure. After confirmation of editing in the bulk culture, clones were isolated by serial limiting dilution and confirmed by Sanger sequencing after PCR amplification from genomic DNA using forward primer 5′-AACACTGACATGGTAAATAACG-3′ and reverse primer 5′-GAATGGTAAACTAGCACATCCTGG-3′ (for A597V) and 5′-GACAGTGGAAATATCTCCGAAAT-3′ (for T648M).

#### cKD constructs and parasite transfections

We used CRISPR-Cas9 to modify the native *Pf*AcAS locus and install the linear pSN054 donor vector (Nasamu et al., 2020) that incorporates a C-terminal V5 and 2xHA-tags, a 10x aptamer array, and the TetR-DOZI expression cassette containing the *blasticidin S deaminase* gene, the reporter gene *Renilla luciferase* (*RLuc*), and the fusion proteins TetR-DOZI (Ganesan et al., 2016). The right homology region (RHR) was PCR amplified and inserted into pSN054 using the I-SceI restriction site. Fragments corresponding to the left homology region (LHR) fused to the re-codonized 3′-end of the gene (bp 2710–2991) as well as the target-specifying guide RNA sequence were synthesized using the BioXP 3200 System (SGI-DNA) and cloned into pSN054 using restriction sites FseI/AsisI and AflII, respectively. Donor vector generation was carried out via Gibson assembly, and the final construct was confirmed by restriction digests and Sanger sequencing. Primers used in this study are listed in the key resources table.

Transfection into Cas9-and T7 RNA polymerase-expressing NF54 parasites was carried out by preloading erythrocytes with the AcAS_pSN054 plasmid as described previously (Deitsch et al., 2001). Briefly, 50–100 μg of purified plasmid DNA were mixed with human red blood cells in 0.2 cm cuvettes and subjected to 8 square wave electroporation pulses of 365 V for 1 ms each, separated by 0.1 s. The DNA preloaded red blood cells were inoculated with schizont-stage parasites (e.g. NF54attB, NF54:pCRISPR) to achieve starting parasitemias ≤1% in RPMI 1640 Complete media. Cultures were maintained in 500 nM anhydrotetracycline (aTc; Sigma-Aldrich 37,919) and 2.5 μg/mL Blasticidin S (RPI Corp B12150–0.1). Emergence of transfectants was monitored via Giemsa smears and RLuc measurements. Clonal parasites were obtained by limiting dilution (Rosario, 1981).

#### Immunoblotting of PfAcAS cKD parasites

*Pf*AcAS conditional knockdown parasites were cultured with (50 nM) and without aTc and proteins were extracted after 72 hr via saponin lysis and resuspension in parasite lysis buffer that consists of 4% SDS and 0.5% Triton X-114 in PBS. Protein extracts were mixed with loading buffer containing sodium dodecyl sulfate (SDS) and dithiothreitol (DTT) and loaded onto Mini-PROTEAN TGX Precast Gels (4–15% gradient) in tris-glycine buffer. Proteins were transferred to a polyvinylidene fluoride (PVDF) membrane using the Mini Trans-Blot Electrophoretic Transfer Cell system according to the manufacturer's instructions and blocked with 100 mg/mL skim milk in TBS/Tween. Membrane-bound proteins were probed with mouse anti-HA (1:3,000; Sigma H3663) and rabbit anti-GAPDH (1:5,000; Abcam AB9485) primary antibodies, and anti-mouse (1:5,000; Thermo Fisher Scientific 62–6520) and anti-rabbit (1:5,000; Cell signaling 7074S) horseradish peroxidase (HRP)-conjugated secondary antibodies. Following incubation in SuperSignal West Pico Chemiluminescent substrate (Thermo Fisher Scientific PI34080), protein blots were imaged and analyzed using the ChemiDoc MP System and Image Lab 5.2.0 (Bio-Rad).

#### Histone acetylation and Western blot assays

The effect of MMV019721 treatment on histone acetylation was tested in wildtype 3D7-A10 and drug-selected 3D7-T648M parasites. Synchronous trophozoite-stage (24-32hr post-invasion) cultures at 3–5% parasitemia and 2% hematocrit in complete medium were exposed to test compounds for 3 hr at 37°C. *Pf*AcAS inhibitors were tested at ∼3, 5 and 10x the 72hr EC_50_ of the compound in wild-type parasites (1, 2.5 and 5 μM for MMV019721 and 325, 540 and 1080 nM for MMV084978 respectively). Trichostatin A (TSA), a non-specific HDAC inhibitor and atovoquone (ATQ) were equally active against wt and mutant parasites and were tested at single concentrations corresponding to three times their EC_50_ (70 nM TSA and 7 nM ATQ). DMSO was tested at 0.1% as the vehicle-only control. Following drug exposure, parasites were isolated via saponin lysis ice-cold 0.1% saponin in phosphate-buffered saline (PBS). Isolated parasites were washed 3x in ice-cold PBS containing protease inhibitor cocktail (cOmplete mini, Roche) and lysed in 1x Laemmli Sample Buffer (Biorad) with 5% beta-mercaptoethanol (Sigma-Aldrich). Samples were heated at 99°C for 10 min before centrifugation. Equal quantities of solute were separated on Protean TGX Precast Gels (4–20% gradient) in Tris-SDS-glycine buffer, transferred to nitrocellulose membrane by iblot (Invitrogen), and blocked for 1h in tris-buffered saline (TBS) with 5% skim milk powder. Blocked membranes were probed with antibodies recognising tetra-acetylated H4 (H4ac4, Sigma-Aldrich 06–866), H4K8ac (CST #2594), H3K9ac (CST #9649) or H3 (CST #4499), at 1:1000 dilution, and *Pf*BiP (Shonhai et al., 2007) (at 1:5000 dilution) in 3% milk powder TBS with 0.1% Tween 20 at 4°C overnight and with 800CW goat α-rabbit IgG (Licor) at 1:10,000 dilution. Membranes were visualised using a Licor Odyssey CLX imaging system and quantified using ImageJ.

#### Growth assay

To assess the growth of *Pf*AcAS conditional knockdown parasites, synchronous ring-stage parasites were cultured in the presence (50 and 3 nM) and absence of aTc and set up in triplicate in a 96-well U-bottom plate (Corning 62,406-121). Luminescence was measured at 0, 72, and 120 hr post-invasion using the Renilla-Glo(R) Luciferase Assay System (Promega E2750) and the GloMax Discover Multimode Microplate Reader (Promega). The luminescence values were normalized to chloroquine-treated (200 nM) samples and results were visualized on a scatterplot using GraphPad Prism (version 8; GraphPad Software).

#### Microscopy

Immunofluorescence assay (IFA) was carried out in solution as described (Tonkin et al., 2004). Cells were incubated with mouse anti-HA primary antibody at 1:500 dilution for an hour followed by incubation with anti-mouse Alexa Fluor 488-conjugated secondary antibody (Cell signaling 4408S) at 1:500 dilution. Cells were washed with PBS containing 0.5 μg/mL of DAPI before mounting onto a microscope slide applied with ProLong Diamond Antifade Mountant (Thermo Fisher Scientific P36965).

Fluorescence microscopy was performed using the GE Healthcare DeltaVision Elite Imaging System at the W.M. Keck Microscopy Facility at the Whitehead Institute and images were processed using ImageJ (Collins, 2007).

#### cKD compound susceptibility assays

Solutions of MMV019721 and MMV084978 were dispensed into 96-well U-bottom plates and serially diluted in complete medium to yield final concentrations ranging from 0.003 to 1 μM and 0.006–1.6 μM, respectively. Synchronous ring-stage, aptamer-regulated *Pf*AcAS and control parasite lines were resuspended in varying aTc concentrations (high = 50 nM, low = 1 nM, and no aTc) and distributed into the drug plate. DMSO carrier and chloroquine (200 nM) wells served as positive and negative controls. Luminescence was measured after 72 hr as described above and EC_50_ values were obtained from corrected dose-response curves using GraphPad Prism.

#### Protein expression and purification

*Generation of PfAcAS bacterial expression plasmid.* The gene encoding *Pf*AcAS (UniProt ID C6KTB4) was codon optimised for expression in *Escherichia coli*, chemically synthesized to contain *NdeI* and *XhoI* restriction sites and cloned by Genscript (https://www.genscript.com) into the expression vector pET15b-TEV, a modified pET15b vector (Novagen) encoding an N-terminal 6X-His tag followed by a tobacco etch virus (TEV) protease cleavage site to produce the bacterial expression clone pET15b-PPT343.

*Generation of PfAcAS baculovirus expression plasmid.* The *Pf*AcAS ORF within pET15b-PPT343 was excised and subcloned into a pFastBac-HT (Invitrogen) vector modified to include *NdeI* and *XhoI* restriction sites. The resulting baculovirus expression clone pFastBac-PPT358 was used as a plasmid template in a single PCR reaction containing mutagenesis primers to produce *Pf*AcAS A597V (pFastBac-PPT409) and *Pf*AcAS T648M (pFastBac-PPT410) mutants. The expression clones pFastBac-PPT358, pFastBac-PPT409 and pFastBac-PPT410 were separately transformed into *E. coli* DH10Bac (Invitrogen), and plated on LB agar plates containing appropriate concentrations of kanamycin, gentamycin, tetracycline, X-gal and IPTG as per the manufacturer's protocol. White colonies were selected and recombinant *Pf*AcAS Bacmid DNA isolated by Maxiprep system (Qiagen).

*Transfection of Sf9 cells with PfAcAS Bacmids.* The recombinant *Pf*AcAS Bacmid DNA was transfected into Sf9 insect cells using Insect GeneJuice Transfection Reagent (Novagen). For transfection, 8 × 10^5^ cells mL^−1^ were seeded in a Nunc 6-well cell culture plate (ThermoFisher Scientific) and allowed to attach for at least 1 hr. The lipid reagent and Bacmid DNA were diluted separately into 500 μL Gibco Sf-900 II SFM media (ThermoFisher Scientific) without antibiotics, combined (1 mL) and incubated for at least 45 min to form transfection mix (lipid–DNA complexes). The supernatant from the settled Sf9 cells was removed and the transfection mix was gently pipetted over the cells. The cells were incubated for 24 hr at 27°C. After 24 hr, 1 mL of Sf-900 II SFM media with antibiotics was gently added to the cells and continued incubation at 27°C for another 6 days. The supernatant containing recombinant baculovirus was harvested as passage 0 (P0) virus stock. 50 mL of exponentially growing Sf9 cells (15 × 10^5^ cells mL^−1^) were infected with 0.125 mL of P0 virus and incubated with shaking at 135 rpm at 27°C for 3 days. The supernatant was P1 virus stock. The above procedure was repeated to prepare P2 virus stock. All recombinant baculovirus stocks were preserved in the dark at 4°C.

*Baculovirus expression of PfAcAS protein in Sf9 cells.* For test expression of wild type *Pf*AcAS, *Pf*AcAS A597V and *Pf*AcAS T648M mutants, 50 mL of exponentially growing Sf9 cells (15 × 10^5^ cells mL^−1^) were grown in three 250 mL Erlenmeyer flasks and infected with recombinant baculovirus by the direct addition of 1%, 3% and 5% (v/v) P2 virus stock. A time course (e.g., 24, 48 and 72 hr) study was performed to optimise expression levels and the time for harvest (i.e., ≥80% cell viability). Samples at each time point were analyzed by SDS-PAGE to check expression level and the three *Pf*AcAS proteins were identified by Western blot analysis using anti-His antibody and HRP-conjugated secondary antibodies/chemiluminescence (Pierce) to detect the N-terminal His-tag in *Pf*AcAS, *Pf*AcAS A597V and *Pf*AcAS T648M. For large scale expression, Sf9 insect cells (15 × 10^5^ cells mL^−1^) were grown in 2L Erlenmeyer flasks, infected directly with 1% (v/v) P2 virus stock and cultured at 27°C with shaking at 135 rpm for 72 hr. Infected Sf9 cells were spun down by centrifugation at 1000*g* for 10 min, washed in 1X PBS buffer and harvested by centrifugation at 1000*g* for 10 min. The cell pellets were stored at −20°C until further use.

*PfAcAS protein purification.* The His-tagged *Pf*AcAS, *Pf*AcAS A597V and *Pf*AcAS T648M were purified by metal affinity chromatography followed by gel filtration chromatography (Figure S4A). Protein purifications were done at room temperature. The Sf9 cell pellets were resuspended in buffer A (20 mM Tris-HCl pH 7.2, 200 mM NaCl) supplemented with DNAse and Proteoloc protease inhibitor cocktail (Expedeon). Cells were lysed using a continuous cell disruptor (Constant System) at 30 kpsi within a pre-cooled chamber. The lysate was cleared by centrifugation at 40,000*g* for 30 min at 4°C and the supernatant filtered through 0.2 μm filter. Cleared lysate was loaded onto a 5 mL HisTrap HP column (GE Healthcare) pre-equilibrated with buffer A. The column was washed in two steps with 10 column volumes of buffer A containing 25 mM and 40 mM Imidazole respectively. Proteins were eluted in a linear gradient of 16-100% buffer B (20 mM Tris-HCl pH 7.2, 200 mM NaCl, 250 mM Imidazole; or 500 mM NaCl, 500 mM Imidazole for *Pf*AcAS A597V) over 20 column volumes. At each purification step, samples were analyzed by SDS-PAGE. The eluted protein was dialyzed against buffer A overnight, concentrated using Jumbosep Centrifugal Device (Pall) and loaded onto a Superdex 200 26/60 gel filtration column (GE Healthcare) pre-equilibrated with buffer A. The peak protein fractions were pooled and concentrated up to 0.5 mg/mL (*Pf*AcAS A597V & *Pf*AcAS T648M) or 10 mg/mL (*Pf*AcAS wt) and stored at −80°C until further use. The purified proteins were analyzed by SDS PAGE (2μg) for densitometry, analytical GF (Superdex 200 increase 10/30) (Cytiva) calibrated with standards (BioRAD) and peptide mass fingerprinting using Mass Spectrometry.

#### Human acetyl-CoA synthetase expression

The gene encoding *Hs*AcAS (cytoplasmic) gene (Genebank AF263614.1) containing an N-terminal His tag and TEV site was synthesized by GenScript and cloned into pCDNA 3.1 vector, using Nhe1 and Xho1 restriction sites. The plasmid was transformed into XL1 cells then a maxiprep was carried out.

A transfection into the Expi293 was carried out as described in the Expi293 Expression System user guide (Thermofisher). Briefly 1 μg/mL of plasmid DNA was transfected per mL of transfection. A total volume of 1000 mL was transfected (2 × 500 mL in 2L disposable vented shake flasks). The cells were harvested by centrifugation at 1000 g for 10 min. The pellets were then washed in PBS then centrifuged again. The pellet was stored at −20°C until purification. The resulting pellet weight was 37g. For lysis 150 mL of 25 mM Bicine pH 7.6/0.1% Triton/150 mM NaCl containing Proteoloc Protease inhibiter EDTA Free (AbCAM) and DNase (Sigma) was added to the pellet. This was then heated at 25°C for 20 min in a water bath to defrost. The cells were resuspended then lysed using a Constant Cell Disrupter (Constant Systems) at 30 KPSI. The lysate was then centrifuged at 40,000 g for 20 min. The supernatant was then diluted 1:3 in 25 mM TRIS pH 8.5/500 mM NaCl/20 mM Imidazole/0.5 mM TCEP (Buffer A) then filtered with a VacuCAP 90 Pf Filter 0.8/0.2 (Pall). The supernatant was loaded at 5 mL/min onto a 5 mL HiTrap His column (Cytiva) on an AKTA Pure system at 2–8°C. A 10CV wash of buffer A was then carried out followed by a 5% Buffer B (A+500 mM Imidazole) wash for 7 CV to remove His rich nonspecific binding proteins. A gradient of 5-50% B over 20CV was used to elute the protein. Following SDS PAGE the peak was pooled and quantified. This showed 35 mgs of the *Hs*AcAS protein. This was dialyzed into 3 x 1L of 25 mM TRIS pH 8.5/150 mM NaCl/10% Glycerol/0.5 mM TCEP(Buffer C) to remove the imidazole. The protein was concentrated using a Macrosep 10kDa Advance concentrator (Pall) to 10.5 mL for loading onto an XK26/60 Superdex 200 Gel Filtration column (Cytiva) at 2 mL/min. The protein eluted as a single monomeric peak (Figure S4B). The protein was then concentrated to 15.5 mg/mL using a Macrosep 10kDa Advance concentrator then snap frozen in liquid nitrogen and stored at −80°C. The final yield was 15.5 mgs from a 1L Transfection. The protein was then analyzed by SDS PAGE (2μg) for densitometry, analytical GF (Superdex 200 increase 10/30) (Cytiva) calibrated with standards (BioRAD), Nanotemper Tycho and peptide mass fingerprinting using Mass Spectrometry.

#### *Pf*AcAS EnzChek™ kinetic measurements

Steady-state kinetic measurements were conducted at room temperature using a PheraStar plate reader (BMG). The first step of the reaction of *Pf*AcAS, which produces inorganic pyrophosphate (PPi) that is converted to two molecules of inorganic phosphate using a pyrophosphatase enzyme, was continuously monitored using the EnzChek Phosphate Assay Kit, which gives an absorbance readout at 360 nm (Figure 4A) (Webb, 1992). As demonstrated by Noy, T. and colleagues, the product AcAMP remains tightly bound to the active site in the absence of CoA (Noy et al., 2014), stopping the reaction, resulting in no changes in the signal for the first step, the rate limiting step (Noy et al., 2014), even in the presence of acetate and ATP. By adding CoA, the reaction rapidly proceeds to the second step resulting in further release of pyrophosphate, making the EnzChek assay a suitable option to continuously monitor the activity of *Pf*AcAS. Typical kinetic assays were carried out in clear, flat-bottom, polystyrene, 384-well plates (Greiner) in an 80 μL reaction volume containing 100 mM HEPES (pH7.4), 100 mM NaCl, 250 mM KCl, 50 mM MgCl_2_, 1 mM DTT, 0.004% Tween 20, 0.5 U/mL pyrophosphatase, 0.1 mM MESG, 0.5 U/mL PNP, 2 mM ATP, 2 mM acetate, 0.5 mM CoA and 5 nM recombinant *Pf*AcAS WT or 10 nM recombinant *Pf*AcAS A597V or T648M. Assays were performed by adding 40 μL of a 2-times concentrated substrate mixture including MESG to all wells, and the reactions started by adding 40 μL of a 2-times concentrated reaction mixture containing all other components. The reactions were carried out for 2 to 4 hr with absorbance readings at 360 nm every two to four minutes. Reaction rates were extracted from the linear phase of activity, usually using 10 to 20 points between times of 30 and 100 min of the reactions. Using a phosphate standard curve obtained under the same buffer conditions used for the *Pf*AcAS reactions (Figure S5A), the rates were then converted to velocity per second, conversion of one nanomolar of phosphate, per nanomolar of enzyme, per second. The substrates *K*_m_ values were determined by varying the concentration of one of the substrates at saturating concentration of the other two substrates. Steady-state data were fitted using the nonlinear, least-squares, curve-fitting programs of Sigma-Plot for Windows, version 14.0. Individual saturation curves were fit to Equation 1

(Equation 1) $$v=\frac{VS}{K+S}$$

where *V* is the maximal velocity, *S* is the substrate concentration and *K* is the Michaelis constant for the substrate (*K*_m_). Individual saturation curves displaying substrate inhibition were fit to Equation 2

(Equation 2) $$v=\frac{VS}{K+S\times \left(1+\frac{S}{K_{s}}\right)}$$

where *K*_s_ is the substrate inhibition constant.

#### *Pf*AcAS mode of inhibition studies

Using the EnzChek assay platform, the IC_50_ values for MMV019721 and MMV084978 were determined at saturating concentrations of all three substrates, 2 mM ATP, 2 mM acetate and 0.5 mM CoA, in a series of 10, one in three dilutions, of the inhibitors. To determine the steady-state inhibition parameters and patterns associated with both inhibitors, *Pf*AcAS activity was studied in the presence of variable concentrations of one substrate, ATP, acetate or CoA, fixed saturating concentration of the other two substrates (acetate and CoA, ATP and CoA, or ATP and acetate) and several fixed concentrations of inhibitor. Assay ready plates containing different concentrations of inhibitor were prepared using an Echo 550 acoustic dispenser (Labcyte Inc.). The assays were carried out as described in the previous section. Inhibition data obtained under saturating concentrations of substrates, and variable concentration of MMV019721 or MMV084978 were fit to Equation 3

(Equation 3) $$y=y_{min}+\frac{y_{max}-y_{min}}{1+{\left(\frac{IC_{50}}{I}\right)}^{n}}$$

where *y*_min_ corresponds to no inhibition and *y*_max_ to maximum inhibition, *IC*_50_ is the concentration of inhibitor necessary to give 50% inhibition and *I* is the inhibitor concentration. For better comparison between both inhibitors and proteins, the *y*_min_ was fixed to zero and the *y*_max_ to 100. Inhibition data showing linear, competitive patterns in double-reciprocal plots were fit to Equation 4

(Equation 4) $$v=\frac{VS}{K\left(1+\frac{I}{K_{i}}\right)+S}$$

where *K*_i_ is the dissociation constant for the enzyme-inhibitor complex. Inhibition data showing linear, mixed inhibition patterns in double-reciprocal plots were fit to Equation 5

(Equation 5) $$v=\frac{VS}{K\left(1+\frac{I}{K_{is}}\right)+S\left(1+\frac{I}{K_{ii}}\right)}$$

where *K*_is_ is the dissociation constant for the free enzyme-inhibitor complex and *K*_ii_ is the dissociation constant for the enzyme-substrate-inhibitor complex. Inhibition data showing linear, uncompetitive patterns in double-reciprocal plots were fit to Equation 6

(Equation 6) $$v=\frac{VS}{K+S\left(1+\frac{I}{K_{i}}\right)}$$

#### *Hs*AcAS rapid-Fire QQQ assays

The human form of acetyl-CoA synthetase was assayed in 100 mM HEPES (pH7.4), 100 mM NaCl, 250 mM KCl, 50 mM MgCl_2_, 1 mM DTT, 0.005% NP40, 400 μM ATP, 70 μM acetate, 40 μM CoA and 2 nM recombinant *Hs*AcAS. Assay ready plates containing different concentrations of inhibitor were prepared using an Echo 550 acoustic dispenser (Labcyte Inc.), dispensing 150 nL of compound per well. Assays were conducted in a total volume of 15 μL for 30 min at room temperature before being quenched with the addition of 85 μL 1% formic acid containing 0.5 μg/mL n-Propionyl coenzyme A (Sigma P5397) as an internal standard. Reaction products were detected using a RapidFire 365 system (Agilent, Santa Clara, CA) coupled with a triple quadrupole mass spectrometer 6740 (Agilent).

The samples were loaded onto a D-hypercarb cartridge (Agilent) using deionized water at a flow rate of 1.5 mL/min and eluted to the mass spectrometer using 5 mM ammonium acetate in deionized water/acetonitrile/acetone (proportions of 2/1/1, v/v) at a flow rate of 1.25 mL/min. The sipper was washed to minimize carryover with 0.1% TFA in deionized water followed by 0.1% TFA in acetonitrile/deionized water (95%/5%, v/v). The sipper was washed to minimize carryover with deionized water followed by acetonitrile. Aspiration time, load/wash time, elution time, and re-equilibration time were set to 600, 3000, 5000, and 500 ms, respectively, with a cycle time of approximately 10 s. The triple-quadrupole mass spectrometer with electrospray ion source was operated in positive multiple reaction monitoring (MRM) mode. The detailed setting for the mass spectrometer parameters was as follows: capillary voltage, 3000 V; gas temperature, 350°C; gas flow, 7 L/min; nebulizer, 40 psi; sheath gas temperature, 300°C′ sheath gas flow, 11 L/min; and nozzle voltage, 1500 V. The MRM transitions for acetyl-CoA as a reaction product were set as 810.1/303.1 & 810.1/428.1 (quantifier & qualifier with dwell time of 50 ms, 100V fragmentor, and collision energies of 30 & 28 eV respectively) and for n-propionyl-CoA as an internal standard as 824.2/317.2 & 824.2/428.0 (quantifier & qualifier with dwell time of 50 ms, 100V fragmentor, and collision energies of 32 & 26 eV respectively). The mass resolution window for both parental and daughter ions was set at as a unit (0.7 Da). Peak areas were integrated, and area ratios of acetyl-CoA to the internal standard (n-propionyl CoA) were used for quantitation. Data were converted to % inhibition relative to the uninhibited enzyme control and fitted to Equation 3.

### Quantification and statistical analysis

Data analyses were performed with GraphPad Prism (v8.0) and R (v3.6.1). Data shown in figures are averages of at least 2 replicates with standard deviation, or are representative results of individual experiments. *p values* were stated in the figure legends. Sample size and statistical tests are also reported in the figure legends.

## Supplemental information

Document S1. Figures S1–S7 and Tables S5 and S6

Table S1. Resistance selections, dose-response phenotypes, and sequencing summary, related to Figure 1

Table S2. Mutation calls for resistance selections, related to Figure 1

Table S3. Denoised log2 copy ratios for sequenced clones from MMV084978 and MMV019721 selections, related to Figure 1

Table S4. Metabolite data, related to Figure 2

Document S2. Article plus supplemental information
