# Supplementary material for: Chemogenomics identifies acetyl-coenzyme A synthetase as a target for malaria treatment and prevention
Source: Cell Chem Biol. 2022 Feb 17;29(2):191–201.e8. doi: 10.1016/j.chembiol.2021.07.010 (PMC8878317; doi:10.1016/j.chembiol.2021.07.010)
Supplement: Document S1. Figures S1–S7 and Tables S5 and S6 [file mmc1.pdf]

Cell Chemical Biology, Volume *29*

Supplemental information

Chemogenomics identifies acetyl-coenzyme A synthetase as a target for malaria treatment and prevention

Robert L. Summers, Charisse Flerida A. Pasaje, Joao P. Pisco, Josefine Striepen, Madeline R. Luth, Krittikorn Kumpornsin, Emma F. Carpenter, Justin T. Munro, De Lin, Andrew Plater, Avinash S. Punekar, Andrew M. Shepherd, Sharon M. Shepherd, Manu Vanaerschot, James M. Murithi, Kelly Rubiano, Aslı Akidil, Sabine Ottilie, Nimisha Mittal, A. Hazel Dilmore, Madalyn Won, Rebecca E.K. Mandt, Kerry McGowen, Edward Owen, Chris Walpole, Manuel Llinás, Marcus C.S. Lee, Elizabeth A. Winzeler, David A. Fidock, Ian H. Gilbert, Dyann F. Wirth, Jacquin C. Niles, Beatriz Baragaña, and Amanda K. Lukens

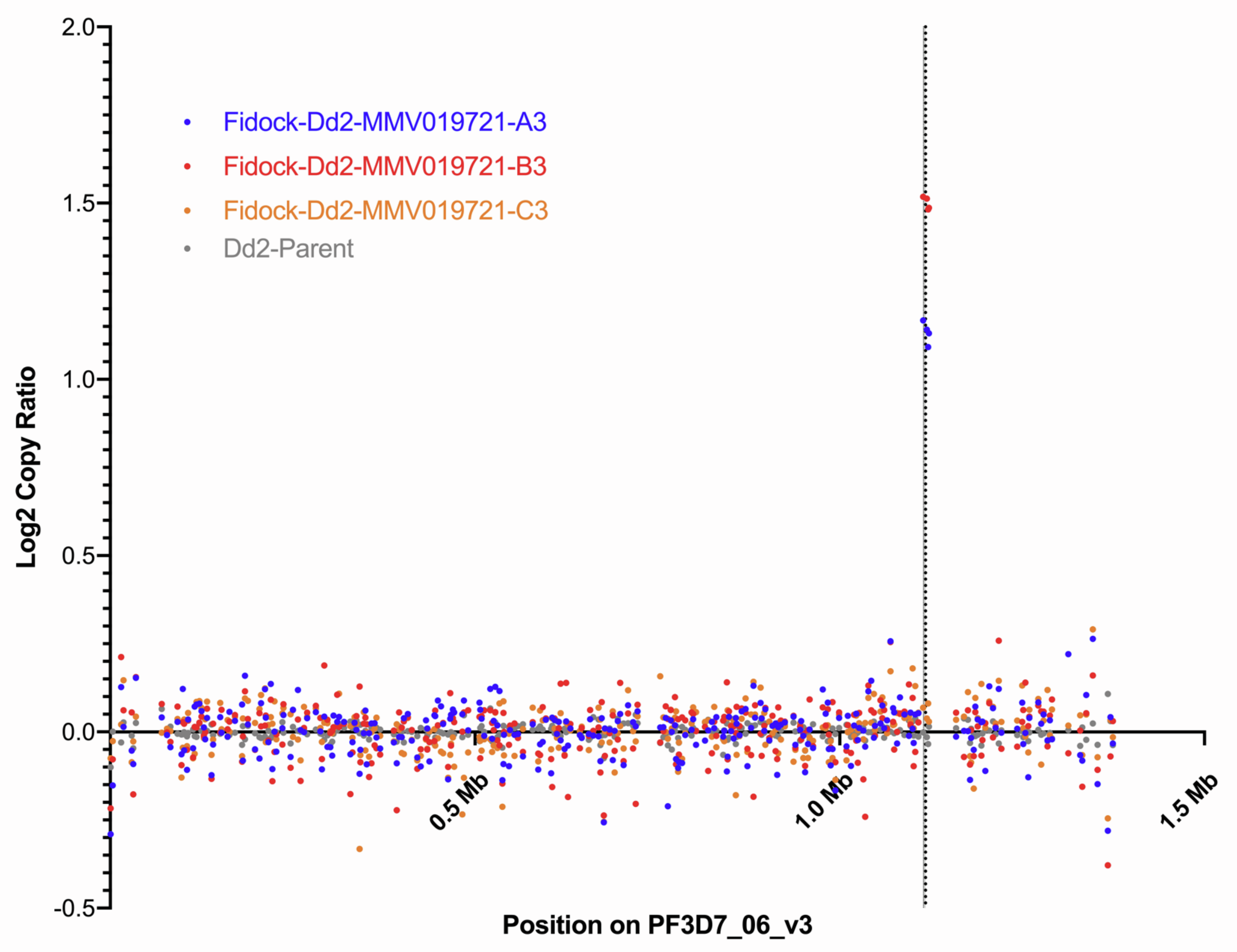

Figure S1, related to Figure 1: Copy number variation in MMV019721-selected lines at the PF3D7_0627800 locus, which encodes PfAcAS. Shown are denoised Log2 copy ratios for gene intervals across chromosome 6 (PF3D7_06_v3) for each sample, calculated using the GATK4 CNV workflow. The dotted line indicates the position of PF3D7_0627800. See also Supplemental Table S3

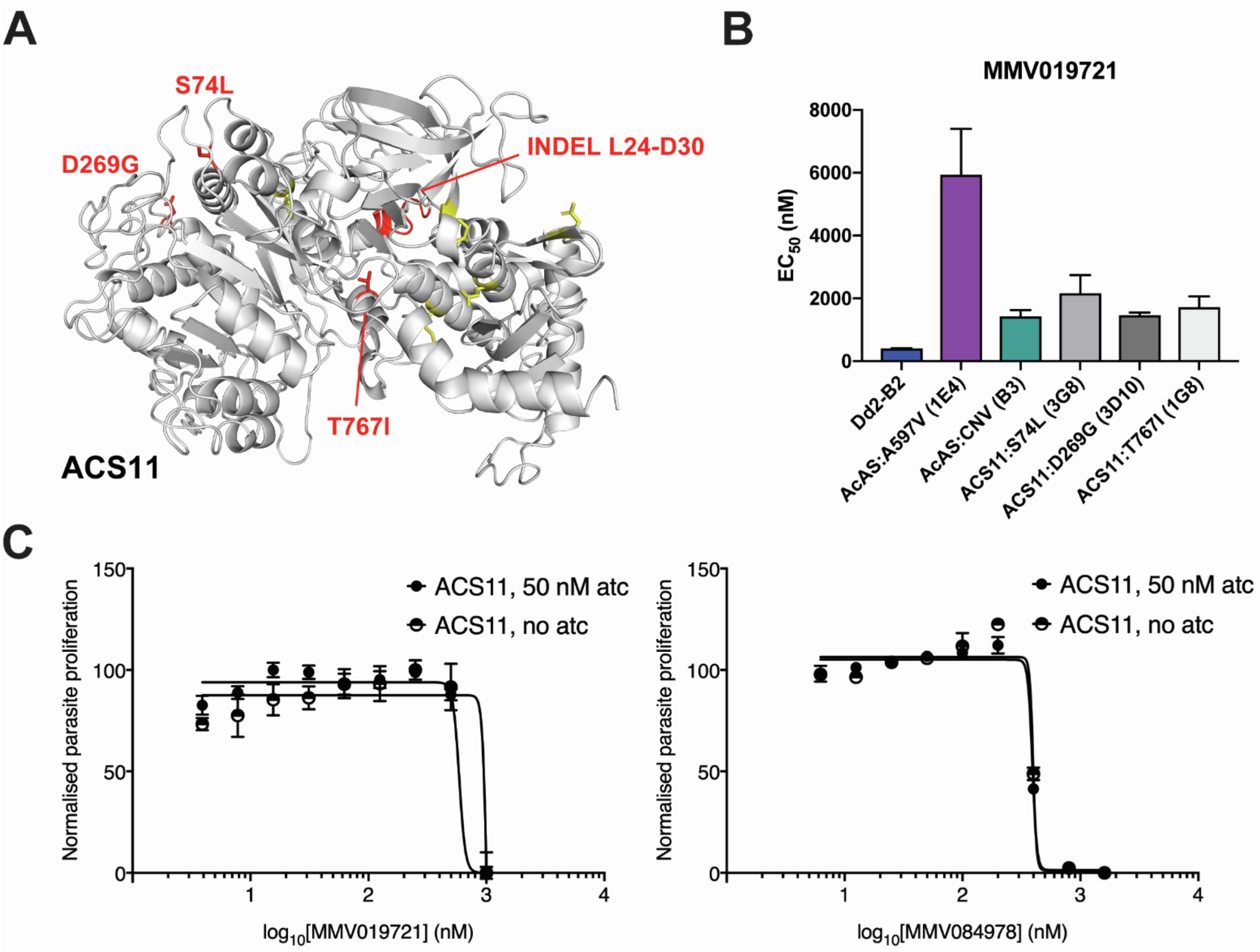

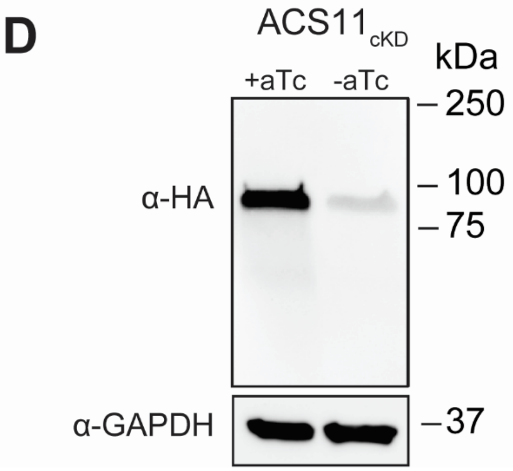

E

	EC50 (nM) ± Std. Dev. (n bioreplicates)		
	PfACS11 cKD (50nM aTc)	PfACS11 cKD (0nM aTc)	fold-shift in EC_{50}
**MMV019721**	560 ± 25 (n=2)	1300 ± 340 (n=2)	+2.4 †
**MMV084978**	390 (n=1)	400 (n=1)	no shift

† "-" indicates leftward shift in cKD condition,
"+" indicates rightward shift in cKD condition

Figure S2, related to Figure 1: The contribution of PfACS11 and PfAcAS copy number variation to altered parasite susceptibility. A) Homology model of *Pf*ACS11 generated by I-TASSER server. Shown in red are mutations identified in parasite clones with low level resistance to MMV019721, while those in yellow arose under selection with pantothenamides (Schalkwijk *et al.*, 2019), and in parasites resistant to MMV019719 or MMV665924 (Cowell *et al.*, 2018). B) EC_{50} values of parasite clones bearing the A597V *Pf*AcAS mutation, amplification of *Pf*AcAS, and *Pf*ACS11 mutations. Shown are mean data ± sd from 2-5 independent experiments. C) Representative effect of *Pf*ACS11 cKD on parasite sensitivity to MMV019721 and MMV084978. D) Western blot of *Pf*AcAS11 protein levels in the presence (50nM) and absence of aTc after 72h. E) EC_{50} values of MMV019721 and MMV084978 for PfACS11 cKD parasites in the presence of high (50nM aTc) and no aTc (0nM aTc). See also Supplemental Table S1.

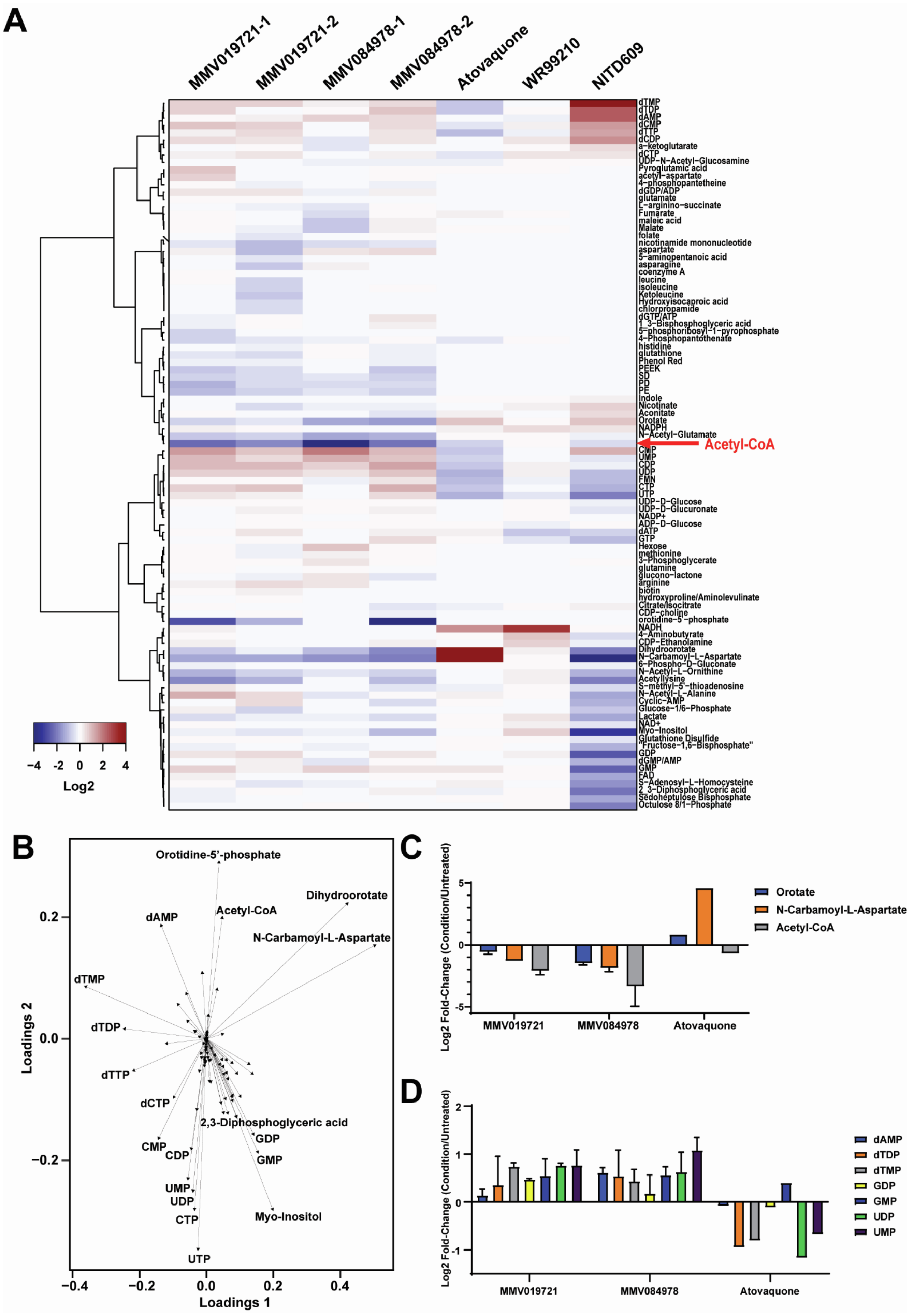

A
MMV019721-1
MMV019721-2
MMV084978-1
MMV084978-2
Atovaquone
WR99210
NITD609
dTMP
dTDP
dAMP
dCMP
dTTP
dCDP
a-ketoglutarate
dCTP
UDP-N-Acetyl-Glucosamine
Pyroglutamic acid
acetyl-aspartate
4-phosphopantetheine
dGDP/ADP
glutamate
L-arginino-succinate
Fumarate
maleic acid
Malate
folate
nicotinamide mononucleotide
aspartate
5-aminopentanoic acid
asparagine
coenzyme A
leucine
isoleucine
Ketoleucine
Hydroxyisocaproic acid
chlorpropamide
dGTP/ATP
1_3-Bisphosphoglyceric acid
5-phosphoribosyl-1-pyrophosphate
4-Phosphopantothenate
histidine
glutathione
Phenol Red
PEEK
SD
PD
PE
Indole
Nicotinate
Aconitate
Orotate
NADPH
N-Acetyl-Glutamate
Acetyl-CoA
CMP
UMP
CDP
UDP
FMN
CTP
UTP
UDP-D-Glucose
UDP-D-Glucuronate
NADP+
ADP-D-Glucose
dATP
GTP
Hexose
methionine
3-Phosphoglycerate
glutamine
glucono-lactone
arginine
biotin
hydroxyproline/Aminolevulinate
Citrate/Isocitrate
CDP-choline
orotidine-5-phosphate
NADH
4-Aminobutyrate
CDP-Ethanolamine
Dihydroorotate
N-Carbamoyl-L-Aspartate
6-Phospho-D-Gluconate
N-Acetyl-L-Ornithine
Acetyllysine
S-methyl-5'-thioadenosine
N-Acetyl-L-Alanine
Cyclic-AMP
Glucose-1/6-Phosphate
Lactate
NAD+
Myo-Inositol
Glutathione Disulfide
"Fructose-1,6-Bisphosphate"
GDP
dGMP/AMP
GMP
FAD
S-Adenosyl-L-Homocysteine
2_3-Diphosphoglyceric acid
Sedoheptulose Bisphosphate
Octulose 8/1-Phosphate
−4 −2 0 2 4
Log2
B
Orotidine-5'-phosphate
Dihydroorotate
dAMP
Acetyl-CoA
N-Carbamoyl-L-Aspartate
dTMP
dTDP
dTTP
dCTP
2,3-Diphosphoglyceric acid
CMP
CDP
GDP
GMP
UMP
UDP
CTP
Myo-Inositol
UTP
0.2
0.0
−0.2
Loadings 2
−0.4
−0.2
0.0
0.2
0.4
Loadings 1
C
Orotate
N-Carbamoyl-L-Aspartate
Acetyl-CoA
5
0
-5
Log2 Fold-Change (Condition/Untreated)
MMV019721
MMV084978
Atovaquone
D
dAMP
dTDP
dTMP
GDP
GMP
UDP
UMP
2
1
0
-1
Log2 Fold-Change (Condition/Untreated)
MMV019721
MMV084978
Atovaquone

Figure S3, related to Figure 2: Effects of MMV019721 and MMV084978 on parasite metabolism. A) Heatmap of Log2 transformed results of treated vs no drug metabolomics comparisons on 98 detected compounds that passed quality control standards. Acetyl-CoA is marked by a red arrow. B) Loadings plot corresponding to PCA analysis (see Figure 1) performed on metabolomics data with different malaria drugs. Labelled compounds correspond to the 18 metabolites with the greatest distance from the origin of the PCA loadings plot. Metaboanalyst was used for the generation of this plot (Chong and Xia, 2018; Chong et al., 2019) C) Decreasing metabolite trends for MMV019721 and MMV084978 involve orotate metabolism as well as acetyl-CoA (averaged, n=2 + sd). D) Trend of increasing nucleotides as a result of MMV019721 or MMV084978 treatment (averaged, n=2 + sd). See also Supplemental Table S4.

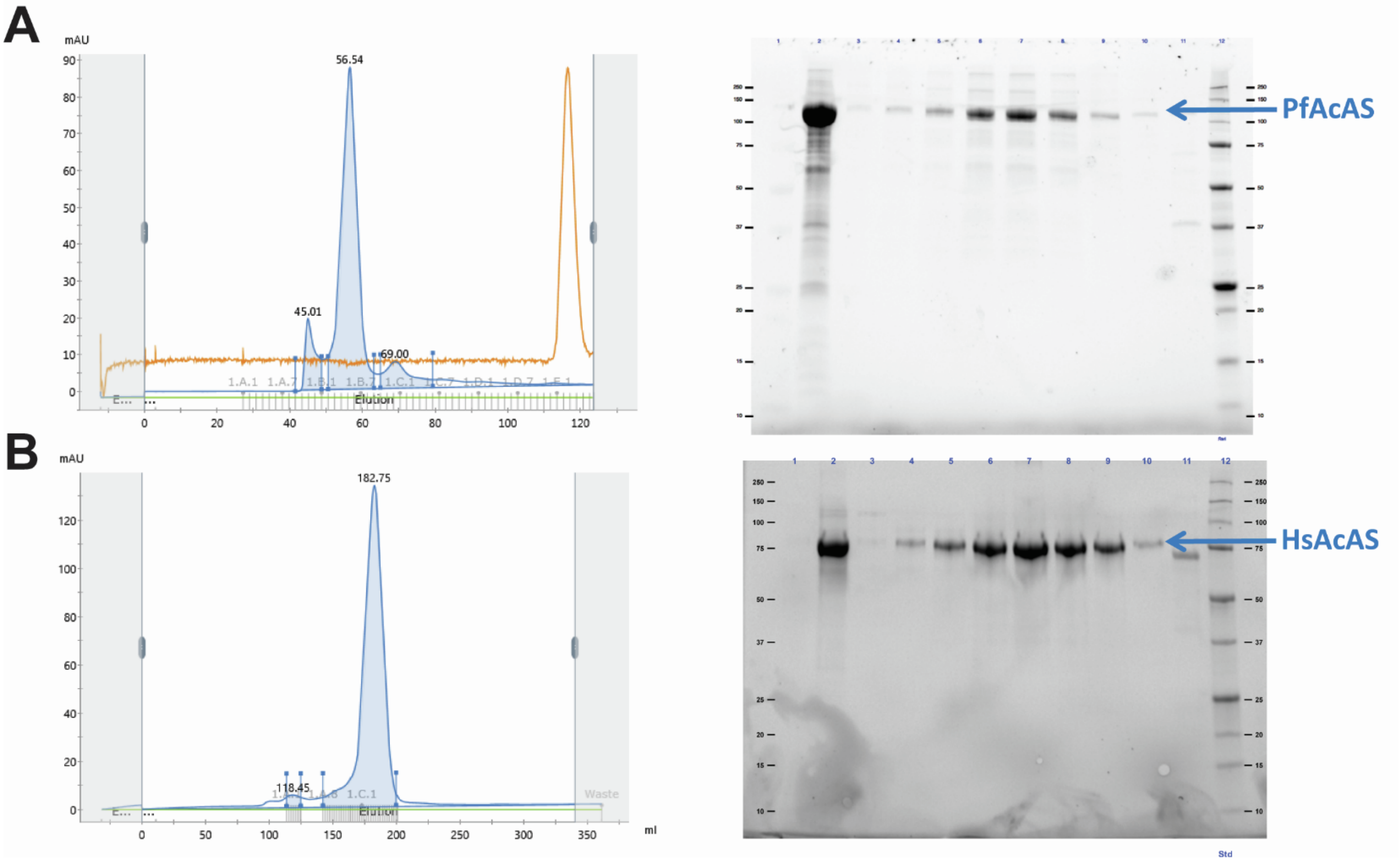

Figure S4, related to Figure 4: Purification of *Pf*AcAS and *Hs*AcAS recombinant proteins. Gel filtration elution profile of (A) full-length *Pf*AcAS and (B) full-length *Hs*AcAS, using a Superdex S200 column. SDS-PAGE gel of Ni-IMAC purified sample (lane 2), peak fractions from the gel filtration column (lanes 3-11) and protein molecular weight marker (lane 12) is reproduced to the right of the chromatograms.

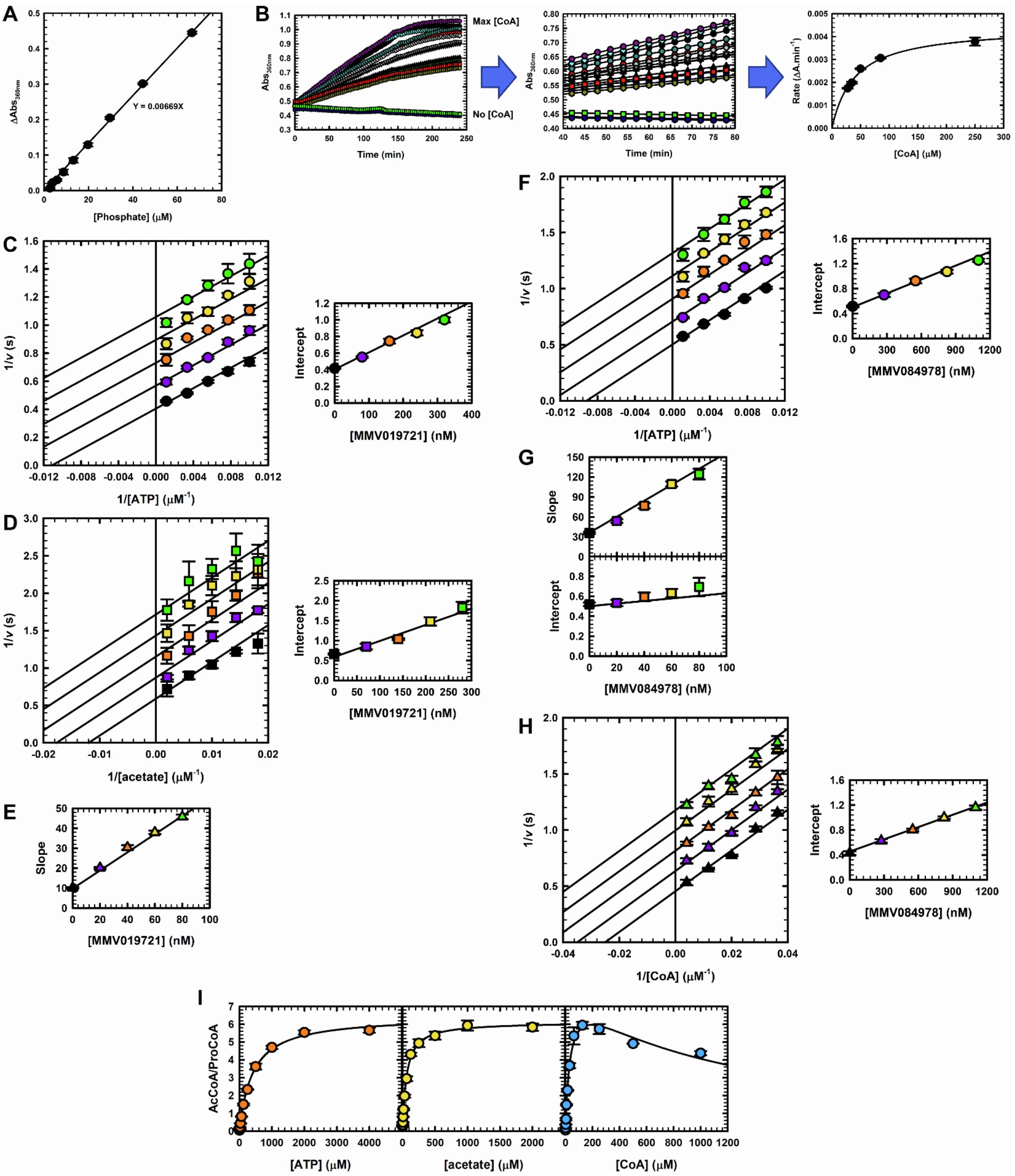

Figure S5, related to Figure 4: Enzymology of *Pf*AcAS and *Hs*AcAS and mode of inhibition studies. (A) EnzChek assay phosphate standard curve. **(B)** EnzChek assay data analysis, varying CoA concentration at saturating concentration of ATP and acetate. Note that in the presence of ATP and acetate, but absence of CoA, the signal does not change, meaning that pyrophosphate is not being formed. This happens because the product AcAMP remains tightly bound to the active site in the

absence of CoA, stopping the reaction. More precisely, the reaction goes through one single turnover producing pyrophosphate, however, because only 5 nM of enzyme is used in the assay, the amount of pyrophosphate produced is lower than the limit of detection of the EnzChek assay. **(C)** Double-reciprocal plot illustrating the linear, uncompetitive inhibition pattern obtained when varying the concentration of MMV019721 at fixed variable concentrations of ATP or **(D)** acetate. For all double-reciprocal plots, points are data obtained with different concentrations of MMV019721 or MMV084978, indicated in the adjacent replot of the data, and error bars indicate the standard deviation (SD). Lines are the best fit of each entire data set to eq. 6. For all replots, points are data, the lines are linear regressions of the data and error bars indicate the SD. **(E)** Replot of the slopes of the data shown in Figure 4C. **(F)** Double-reciprocal plot illustrating the linear, uncompetitive inhibition pattern obtained when varying the concentration of MMV084978 at fixed variable concentrations of ATP or **(H)** CoA. **(G)** Replots of the slopes (top panel) and intercepts (bottom panel) of the data shown in Figure 4D. Shown are the mean ± standard deviation of the results of three independent experiments. **(I)** HsAcAS steady-state kinetics. Saturation curves for ATP (orange), acetate (yellow) and CoA (blue). Shown are the mean ± standard deviation of the results of three independent experiments. Lines are the best fit to equations 1 (ATP and acetate) and 2 (CoA) in methods. See also Supplemental Table S5, S6.

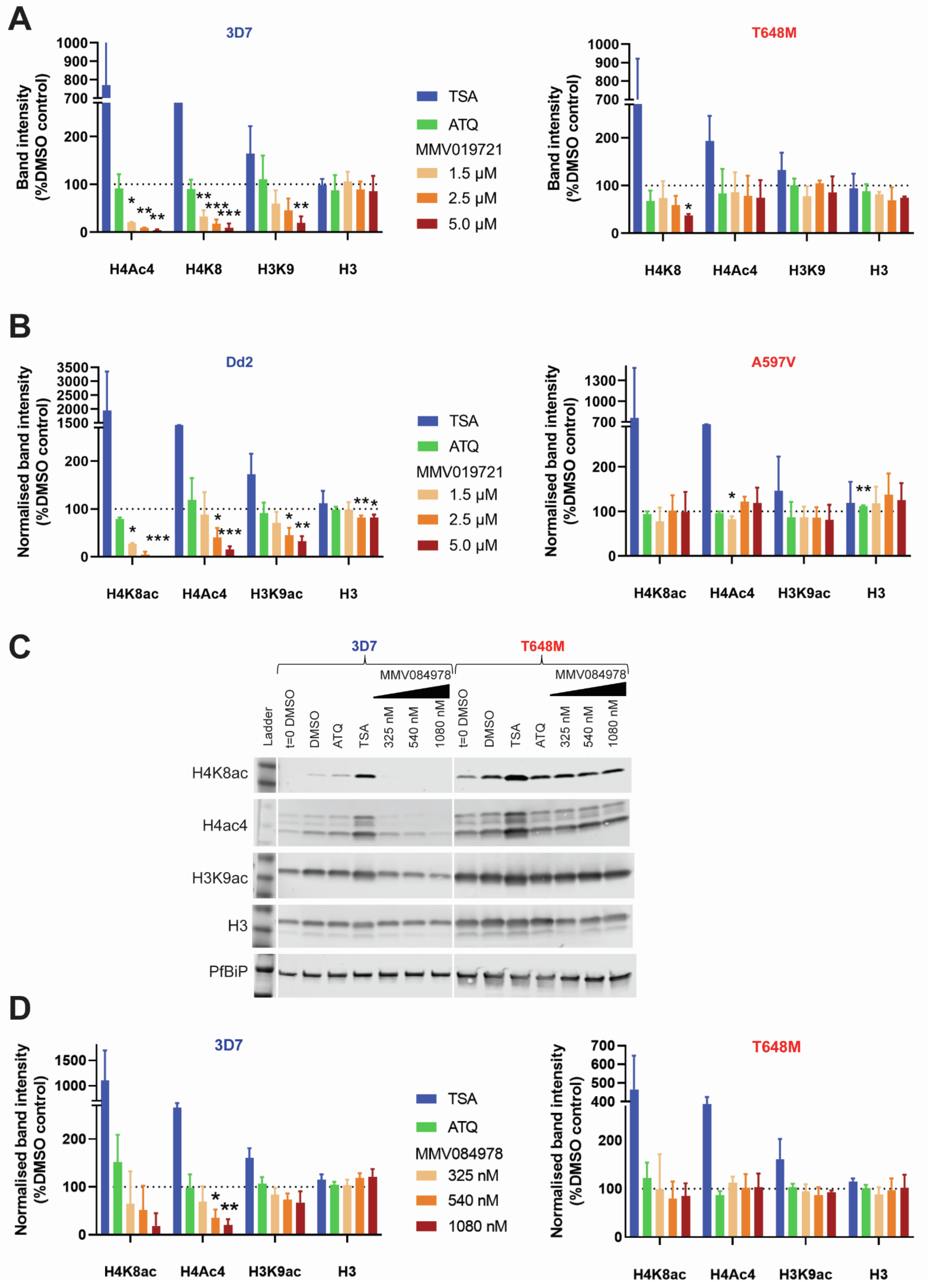

A
3D7
Band intensity (%DMSO control)
1000
900
800
700
200
100
0
H4Ac4
H4K8
H3K9
H3
TSA
ATQ
MMV019721
1.5 µM
2.5 µM
5.0 µM
T648M
H4K8
H4Ac4
H3K9
H3
B
Dd2
Normalised band intensity (%DMSO control)
3500
3000
2500
2000
1500
H4K8ac
H4Ac4
H3K9ac
H3
A597V
1300
1000
700
C
3D7
T648M
MMV084978
Ladder
t=0 DMSO
DMSO
ATQ
TSA
325 nM
540 nM
1080 nM
H4K8ac
H4ac4
H3K9ac
H3
PfBiP
D
3D7
1500
1000
325 nM
540 nM
1080 nM
T648M
700
600
500
400

Figure S6, related to Figure 5: Influence of MMV019721 and MMV084978 on histone acetylation. (A) Dose-dependent reduction of histone acetylation by MMV019721 in wildtype 3D7 parasites but not in *Pf*AcAS-T648M carrying drug resistant parasites. (B) Dose-dependent reduction of histone acetylation by MMV019721 in wildtype Dd2 parasites but not in *Pf*AcAS-A597V carrying drug resistant parasites. Band intensity of acetyl-histone markers was quantified using ImageJ and expressed as a percentage of the DMSO control. Shown are the mean + standard deviation of the results of four independent experiments. (C) Dose-dependent reduction of histone acetylation by MMV084978 in wildtype 3D7 parasites but not in PfAcAS-T648M carrying drug resistant parasites. Shown are representative western blots of a single experiment. (D) Band intensity of acetyl-histone markers was quantified using ImageJ and expressed as a percentage of the DMSO control. Shown are the mean + standard deviation of the results of four independent experiments. The asterisks denote a significant difference in the band intensity of histone markers under compound treatment compared to the DMSO control: *** $P < 0.001$, ** $P < 0.01$, * $P < 0.05$ (Dunnett's multiple comparison tests).

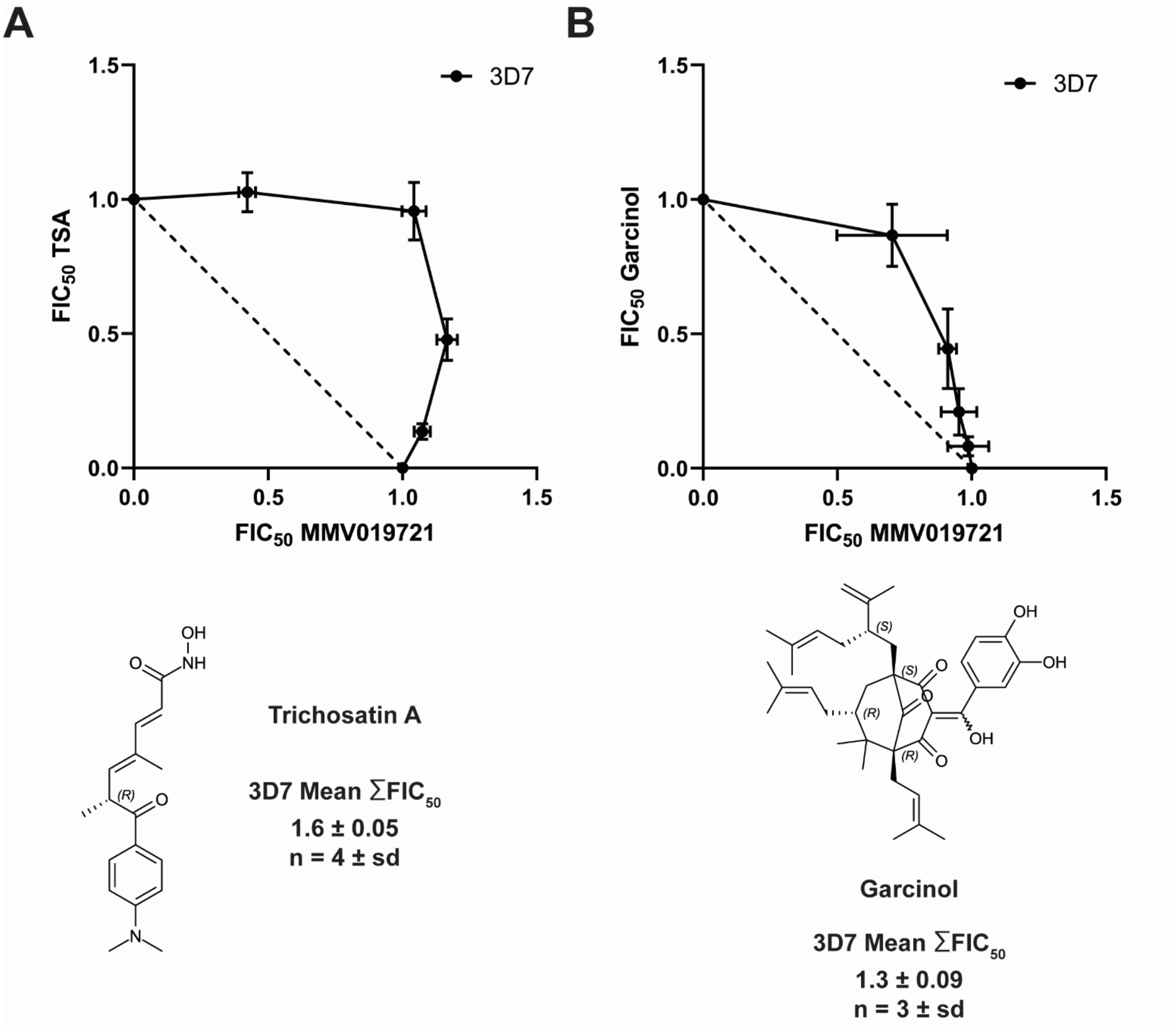

Figure S7, related to Figure 5: Antagonistic interactions between MMV019721 and histone acetylation modulators TSA (HDAC inhibitor) and Garcinol (Histone acetyltransferase inhibitor). Data are shown as the mean ± standard deviation in the fractional IC_{50} value (FIC_{50}) of each compound combination, averaged from 3-4 independent experiments, within which treatments were performed in triplicate.

Table S5, related to Figure 4: Steady-state kinetic parameters for PfAcAS WT, T648M and A597V, and HsAcAS WT. Values presented are mean ± standard deviation.

Substrate	AcAS	Vmax (s-1)	Km (µM)	Ki (µM)
ATP	PfWT	2.4 ± 0.03	93 ± 4	-
	T648M	1.9 ± 0.03	400 ± 17	-
	A597V	0.54 ± 0.01	140 ± 5	-
	HsWT	N/A[a]	400 ± 22	-
Acetate	WT	2.2 ± 0.04	72 ± 5	-
	T648M	1.6 ± 0.02	190 ± 8	-
	A597V	0.54 ± 0.05	400 ± 12	-
	HsWT	N/A[a]	61 ± 4	-
CoA	WT	2.7 ± 0.08	42 ± 3	2700 ± 310
	T648M	1.9 ± 0.05	82 ± 6	4200 ± 450
	A597V	0.84 ± 0.05	61 ± 6	370 ± 40
	HsWT	N/A[a]	36 ± 5	1000 ± 190

[a] N/A = Not applicable; In the case of HsWT, the assay used reports on the correlation levels between AcCoA and ProCoA, and therefore the Vmax is not expressed in a unit of time.

Table S6, related to Figure 4: Steady-state kinetic parameters for the mode of inhibition (MoI) of PfAcAS WT by MMV019721 and MMV084978. Values presented are mean ± standard deviation.

Parameter	MMV019721 Best fit	MMV019721 MoI[a]	MMV019721 Best fit	MMV019721 MoI[a]
**Vmax,ATP (s-1)**	2.5 ± 0.05	-	2.0 ± 0.04	-
**Km,ATP (µM)**	90 ± 5	-	110 ± 6	-
**Ki,ATP (nM)**	200 ± 8	UC[b]	680 ± 26	UC[b]
**Vmax,acetate (s-1)**	1.7 ± 0.06	-	2.0 ± 0.04	-
**Km,acetate (µM)**	84 ± 8	-	73 ± 5	-
**Kis,acetate (nM)**	150 ± 10	UC[b]	30 ± 3	MI[c]
**Kii,acetate (nM)**	-	-	390 ± 110	MI[c]
**Vmax,CoA (s-1)**	2.4 ± 0.02	-	2.2 ± 0.04	-
**Km,CoA (µM)**	23 ± 1	-	40 ± 2	-
**Ki,CoA (nM)**	22 ± 1	C[d]	690 ± 24	UC[b]

[a] MoI = Mode of Inhibition
[b] UC = uncompetitive
[c] MI = mixed inhibition
[d] C = competitive
